# *Humulus lupulus* (Hop)-Derived Chemical Compounds Present Antiproliferative Activity on Various Cancer Cell Types: A Meta-Regression Based Panoramic Meta-Analysis

**DOI:** 10.3390/ph18081139

**Published:** 2025-07-31

**Authors:** Georgios Tsionkis, Elisavet M. Andronidou, Panagiota I. Kontou, Ioannis A. Tamposis, Konstantinos Tegopoulos, Panagiotis Pergantas, Maria E. Grigoriou, George Skavdis, Pantelis G. Bagos, Georgia G. Braliou

**Affiliations:** 1 Department of Computer Science and Biomedical Informatics, University of Thessaly, 35131 Lamia, Greece; tsiogeorgios@uth.gr (G.T.); eandroni@uth.gr (E.M.A.); itamposis@uth.gr (I.A.T.); pbagos@compgen.org (P.G.B.); 2 Department of Mathematics, University of Thessaly, 35132 Lamia, Greece; pkontou@uth.gr; 3 Department of Molecular Biology & Genetics, Democritus University of Thrace, 68100 Alexandroupolis, Greece; ntinosteg13@gmail.com (K.T.); mgrigor@mbg.duth.gr (M.E.G.); gskavdis@mbg.duth.gr (G.S.); 4 Bioapplications Ltd., 32131 Levadia, Greece; pergantas@bioapplications.gr

**Keywords:** *Humulus lupulus*, hop, antiproliferative, anticancer, meta-analysis, meta-regression

## Abstract

**Background/Objectives**: *Humulus lupulus* (hops) are a perennial, dioecious plant widely cultivated for beer production, used for their distinguishing aroma and bitterness—traits that confer high added value status. Various hop-derived compounds have been reported to exhibit antioxidant, antimicrobial, antiproliferative and other bioactive effects. This systematic review and meta-analysis assesses the impact of hop compounds on the viability of diverse cancer cell lines. **Methods**: A comprehensive literature search was performed following PRISMA guidelines. Data were synthesized via multivariate meta-analysis and meta-regression, using IC_50_ values as the effect size. Key variables included assay type (SRB, tetrazolium salt-based, crystal violet), exposure duration (24, 48, 72 h), specific hop compound and cancer cell line. **Results**: Of 622 articles identified, 61 met eligibility criteria, yielding 354 individual experiments. Meta-regression of xanthohumol (XN) IC_50_ values across SRB, tetrazolium and crystal violet assays revealed no statistically significant differences at 24 h (*p* = 0.77), 48 h (*p* = 0.35) and 72 h (*p* = 0.70), supporting the interchangeability of the methods. Meta-analysis confirmed that hop constituents inhibit cancer cell proliferation; XN emerged as the most potent flavonoid (IC_50_ = 16.89 μM at 72 h), while lupulone was the strongest compound overall (IC_50_ = 5.00 μM at 72 h). Crude hop extracts demonstrated greater antiproliferative selectivity for cancer versus non-cancer cells (IC_50_ = 35.23 vs. 43.80 μg/mL at 72 h). **Conclusions**: Hop compounds, and particularly bitter acids, demonstrate promising antiproliferative activity against cancer cells with comparatively low toxicity to healthy cells. Furthermore, our analysis confirms the comparability of SRB, tetrazolium-based and crystal violet assays, supporting the robust integration of antiproliferative data.

## 1. Introduction

*Humulus lupulus*, commonly known as hops, are a climbing, flowering, dioecious plant that belongs to the hemp family Cannabaceae. Hops hold significant importance in the brewing industry since its female flowers, also called cones or hops, convey distinctive aromas, flavors and bitterness to the beer, while also inhibiting the growth of beer-spoilage bacteria. Beyond brewing, hops have attracted considerable scientific interest due to a multitude of biological activities, such as antioxidant, cancer chemoprotective, anti-angiogenesis, sedative, antibacterial, estrogenic, antidepressive, antifungal, insect antifeedant and antiviral effects [1,2,3,4,5,6,7,8]. The earliest scientific report of hop constituents goes back to 1913 [9], where its polyphenols, bitter acids and essential oils were first identified.

Xanthohumol (XN) is the most prevalent compound among hop polyphenols constituting 80–90% of all hop prenylflavonoids [10]. XN’s structural isomers, isoxanthohumol (IXN) and desmethylxanthohumol (DMX), and downstream metabolites, including 8-prenylnaringenin (8-PN) and 6-prenylnaringenin (6-PN), comprise much of the remaining prenylflavonoid constituent. These compounds are often present at higher concentrations in beer and within the human intestine because XN is extensively isomerized and metabolized to these derivatives [11]. Apart from polyphenols, bitter acids (prenyl derivatives of floroglucin) represent another key class of hop constituents. Alpha acids encompass humulone and its analogues, while beta acids include lupulone and its analogues [12].

Xanthohumol has been reported to show potential “broad-spectrum” anticancer activity in the initiation, promotion and progression of cancer [13]. Several reports on various carcinoma cell lines suggest a range of mechanisms through which XN may exert its anticancer potential. Cancer chemo-prevention activity has been attributed to its antioxidant activity by scavenging ROS, thus preventing oxidative damage [3,14]. In addition, the anticancer activity of XN may be mediated through its apoptotic potential by inducing caspase-dependent degradation of the BCR-ABL oncoprotein [15] or via activation of the MAPK JNK kinase [16]. It has also been proposed that XN anticancer activity involves the inhibition of DNA synthesis or G0/G1 cell cycle arrest [12].

Hops’ alpha bitter acids possess mild antiseptic activity against Gram-positive bacteria while their beta acids are reported to be more potent antimicrobials [12,17], thus making hops particularly suitable as beer preservatives [18]. Moreover, alpha bitter acids exhibit sedative and hypnotic effects with potential therapeutic applications in insomnia and attention deficit hyperactivity disorder (ADHD) through modulation of the GABAA receptors. Beta bitter acids, on the other hand, have been associated with antidepressant-like effects [19,20]. While there is substantial evidence that XN possesses cancer antiproliferative activity, only a limited number of studies have examined the antiproliferative activity of hop bitter acids; notably, these studies suggest that beta bitter acids may exhibit substantially greater antiproliferative activity than XN [17].

Given the wide variety of hop compounds and extract types tested across numerous cancerous and non-cancerous cell lines, it becomes imperative to quantitatively assess and compare the antiproliferative effects of each individual compound and extract. The variation in treatment protocols—particularly in compound concentrations and incubation times—adds an additional layer of complexity when comparing results across cell culture studies. These inconsistencies make it difficult to reliably assess and compare the antiproliferative efficacy of different hop compounds against various cancer cell lines.

Another parameter that further shapes the controversy of the antiproliferative effect is the wide range of assays used to measure cell viability. Broadly, these assays fall into three main categories: (a) sulforhodamine B (SRB) assays that measure cellular protein content [21], (b) tetrazolium dye assays (MTT) based on NAD(P)H-dependent cellular oxidoreductase enzymes [22] and (c) a crystal violet assay (CV), which quantifies DNA as a proxy for a cell number [23]. The outcomes of these assays are typically reported as IC_50_ values, i.e., the concentration of a compound needed to inhibit a biological process (herein cell viability or proliferation) by 50%.

The emerging interest in specific natural compounds and medicinal plant extracts with potential health benefits underscores the need to systematically synthesize, compare and summarize antiproliferative activity data of hop-derived compounds. This can be effectively achieved through a systematic review and meta-analysis, ensuring statistical rigor and comprehensive evaluation. Meta-analysis is a component of evidence-based scientific practice, also known as metascience. This approach applies scientific methodologies to analyze scientific results, often from multiple resources, aiming to uncover methodological flaws, inefficiencies and suboptimal practices across various scientific disciplines. Findings from such analyses often serve as valuable guidelines to enhance reproducibility, particularly in biomedical research, by integrating results across diverse scientific methodologies and protocols. The field of metascience has emerged in response to the so-called “replication crisis,” reflecting a growing awareness of the need for greater transparency, rigor and reliability in scientific research [24,25,26].

The objective of this study is to statistically synthesize all available data from the literature to evaluate the antiproliferative potential of various hop compounds across a broad spectrum of cancer cell lines. This meta-analysis aims to quantitatively integrate available evidence, identify interchangeable methodologies and summarize the antiproliferative effects of the studied compounds. By analyzing diverse study outcomes, it seeks to provide a comprehensive overview that extends beyond the insights of individual studies.

Through meta-regression, the analysis also investigates methodological equivalence and, given the differing chemical principles underlying various assays, assesses the feasibility of establishing more reliable approaches for cross-study comparisons.

## 2. Results

### 2.1. Selection and Characteristics of Studies

**Figure 1:**
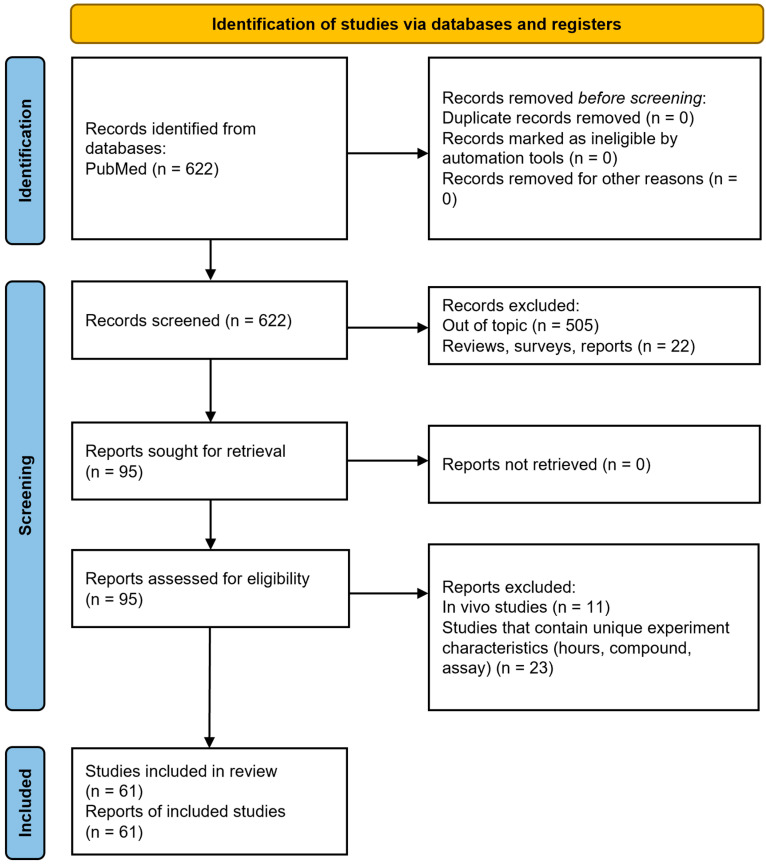
PRISMA-compliant flow diagram of systematic review to retrieve selected studies for present systematic review and meta-analysis.

**Figure 2:**
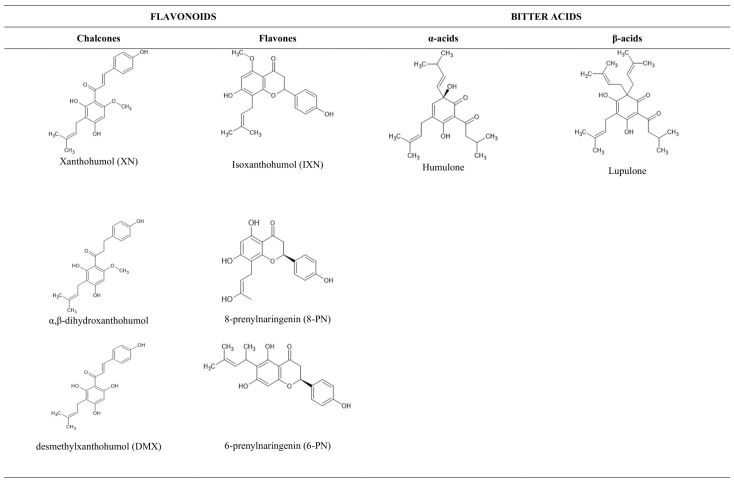
The main flavonoids and bitter acids from *Humulus lupulus*, studied herein.

From the systematic literature search in the PubMed database carried out by 1 June 2024, according to PRISMA guidelines, 622 articles were retrieved. Of these, 561 were excluded because they did not provide data (studies were on extracts of other plants, other biological parameters or reviews) (Figure 1). The 61 articles (encompassing cell viability assays for hop compounds) that fulfilled the eligibility criteria incorporated data from a total of 317 experiments on various cell lines that were included in the meta-analysis. There were 262 experiments involving cancer cell lines, comprising 19 different cancer types and 14 chemical compounds (Table 1 and Supplementary Table S1), with breast cancer being the most common. There were 55 experiments on normal cell lines. Of the eight chemical compounds tested in the 262 experiments (Figure 2), XN was the most studied (in 55% of the total experiments), IXN was studied in 15% of the experiments, 8-prenylnaringenin in 9%, α,β-dihydroxanthohumol in 4%, DMX in 4% and 6-prenylnaringenin in 4%. Moreover, 153 experiments were performed with tetrazolium salt-based assays, 139 with the SRB assay and 25 with the CV assay. The MTT, XTT, WST or MTS assays were all grouped within the tetrazolium salt-based assays (tetrazolium), since they are all based on the same chemical principle, in order to achieve a higher-order classification group and to obtain a more statistically powerful pooled effect size. Our meta-analysis was also built on the basis of the same time period of cells exposed to compounds, thus creating three main groups of 24, 48 and 72 h of treatment.

### 2.2. Interchangeability of Tetrazolium-Based, SRB and CV Assays

**Figure 3:**
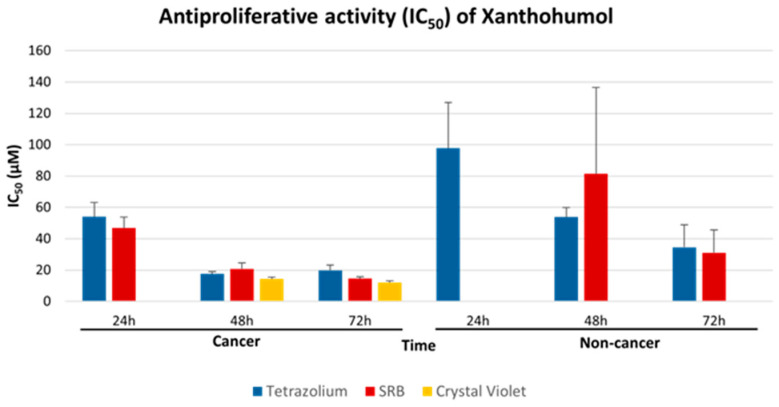
Meta-analysis of IC_50_ values (and SE) of xanthohumol (ΧΝ) on various cell types, for different incubation times, obtained from tetrazolium salt, SRB and crystal violet (CV) assays.

A great concern of researchers investigating antiproliferative activity of plant extracts is which type of assay best corresponds to the true values. In the absence of a gold standard method, many investigators perform the same experiments using two or three of the tetrazolium salt-based, SRB and CV assays to validate their findings and ensure reliability. Initial meta-analysis stratifying for every compound, every incubation time period and every cell type (plus collectively for cancer and non-cancer cell lines), along with stratification for each type of assay, produced such an overabundance of results regarding IC_50_ values (Supplementary Table S2) that analyzing each of these contrasts would produce puzzling results that are difficult to interpret. In the present study, we took advantage of the plethora of experiments available on ΧΝ activity to test whether any of these assays could be used interchangeably. The trigger towards this idea was given by the fact that the IC_50_ values for ΧΝ for all time points (24, 48, 72 h) during the treatment of cancer cell lines were quite close between all three methods. As shown in Figure 3 (and Supplementary Table S3) for cancer cell lines after 48 h, the IC_50_ values were 17.64 μM, 20.78μM and 14.38 μM, while after 72 h, they were 19.85 μM, 14.60 μM and 12.06 μM, for the tetrazolium salt, SRB and CV assays, respectively. Similarly, for non-cancer cells, the respective IC_50_ values were, after 48 h, 53.89 μM for tetrazolium, and after 72 h, 34.53 μM and 31.03 μM for the tetrazolium and SRB assays, respectively. No data existed for non-cancer cells with the CV assay.

To test the hypothesis of whether these differences in IC_50_ values are due to statistically significant variations, a meta-regression-based meta-analysis was employed to explore the extent to which statistical heterogeneity between results from multiple studies may be related to differences inherent to the assays. As shown in Table 2 (and Supplementary Figure S1), the *p*-values of all contrasts are >0.05 (*p*-values 0.78, 0.35 and 0.70 for 24 h, 48 h and 72 h for cancer cells, and 0.52, 0.36 and 0.91 for the non-cancer cells—with no data available from the CV method on non-cancer cells), suggesting that indeed, the IC_50_ values obtained by each method for cancer and non-cancer cell lines, at each time point of incubation, do not differ statistically significantly. Therefore, these three methods provide comparable results and can be used equivalently to quantify cell viability.

To further verify the equivalence of the tetrazolium salt, SRB and CV methods, a meta-analysis employing standardized mean difference (SMD) approach was performed for studies assessing the same compound outcome on the same cell line for the same incubation time, with results derived from two different assays (Supplementary Table S4). As shown in Table 3, the SMDs of IC_50_ values derived from tetrazolium salt and CV assays, on exactly the same cell lines, do not show statistically significant differences (SMDs), corroborating the notion that these methods provide equivalent results.

### 2.3. Antiproliferative Effect of XN Increases with Time of Incubation

**Figure 4:**
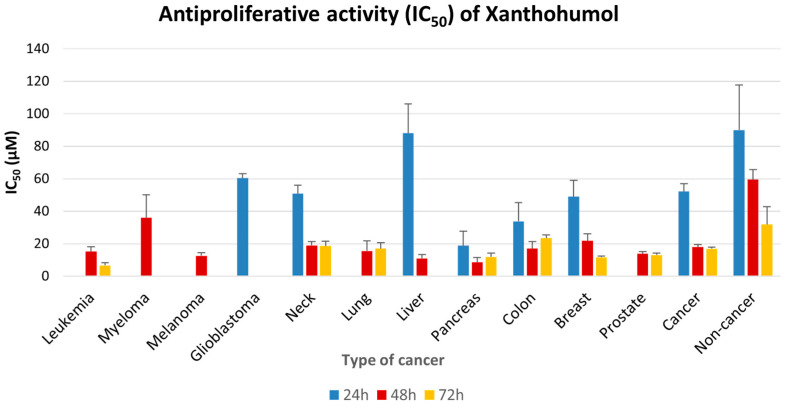
Meta-analysis of IC_50_ values (and SE) of XN on different cancer types and non-cancer cells for different incubation times, obtained from all assays, collectively.

Next, and after having proven the equivalence of the three methods, a comprehensive meta-analysis was employed, incorporating the results of the XN treatment obtained via all three methods. Breast and prostate cancer cell lines were the most frequently studied (Supplementary Table S5). Meta-analysis showed that the IC_50_ value of XN after 72 h in breast cancer is 11.60 μM (13 studies), and in prostate cancer 13.0 μM (9 studies). As shown in Figure 4 and Supplementary Figure S2, the IC_50_ values of XN in all cell lines decrease with time of incubation. Importantly, the respective IC_50_ values for cancer cell lines are lower than those of non-cancer cells, indicating that XN exhibits a more potent antiproliferative effect on cancer cells compared to healthy cells (Supplementary Table S5).

**Figure 5:**
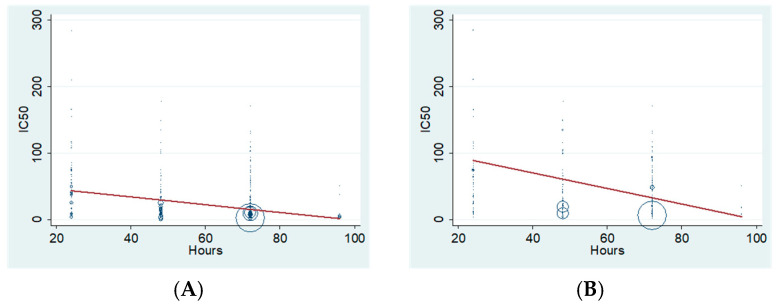

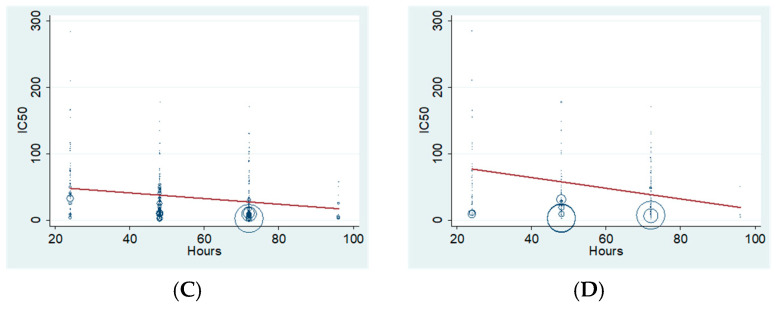
Meta-regression analysis of IC_50_ values (obtained from tetrazolium salt, SRB and CV assays) of (**A**) XN added for different incubation times (24, 48, 72 and 96 h) on all cancer cell lines, collectively; (**B**) XN added for different incubation times (24, 48, 72 and 96 h) on non-cancer cells; (**C**) all hop chemical compounds, jointly, added for different incubation times (24, 48, 72 and 96 h) on all cancer cell lines, collectively; (**D**) all hop chemical compounds, jointly, added for different incubation times (24, 48, 72 and 96 h) on non-cancer cells. Each circle represents an individual study, with its size proportional to the inverse of the variance of the corresponding IC_50_ estimate. The red line represents the fitted meta-regression line, indicating the estimated relationship between incubation time and IC_50_ values.

To further verify the dependence of the IC_50_ values of a certain compound on the incubation time, we next performed a regression meta-analysis. Contrasts were performed for cancer and non-cancer cell lines incubated not only with XN, but also with all of the compounds, collectively, tested in the present study, for 24, 48, 72 and 96 h. As shown in Figure 5 (and Supplementary Table S6), the *p*-values of all contrasts are <0.05, suggesting a clear association of compound incubation time with IC_50_ values. However, separate tests for IXN, 8-PN, 6-PN and lupulone verified the time dependence of the IC_50_ values for 8-PN only.

### 2.4. Antiproliferative Potential of Hops Flavonoids, Bitter Acids and Crude Extracts

**Figure 6:**
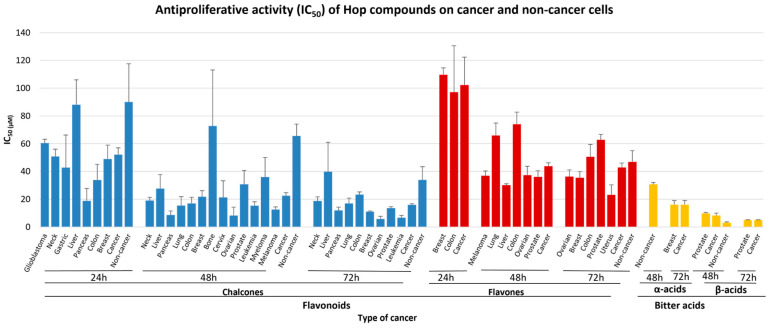
Meta-analysis of IC_50_ values (and SE) of various classes of hop compounds on cancer and non-cancer cells, obtained from tetrazolium salt, SRB and CV assays, for different incubation times. Results are shown for chalcones (blue), flavones (red) and bitter acids (yellow).

To get a better insight into the effect of all studied hop compounds on the proliferation of all tested cell lines, we grouped them into flavonoids (chalcones and flavones) and bitter acids (alpha and beta-acids) and performed a stratification meta-analysis according to cell type and time of incubation (Table 4). We found that chalcones exert a more robust antiproliferative activity on cancer cell lines compared to flavones for all time points i.e., IC_50_ values of 52.16 μM, 22.54 μM and 15.93 μM for 24, 48 and 72 h, respectively, as compared to 102.26 μM, 43.76 μM and 42.95 μM for 24, 48 and 72 h, respectively (Supplementary Figure S3). Another interesting finding is that bitter acids, and especially beta acids, exert even more vigorous antiproliferative activity compared to all flavonoids; i.e., after 72 h of treatment of cancer cell lines, the IC_50_ values were 10.06 μM and 5.00 μM for the alpha and beta acids, respectively (Figure 6 and Table 4). Importantly, the IC_50_ values of the above flavonoids in non-cancer cells were higher compared to cancer cells, suggesting a selective antiproliferative effect against cancer cells compared to non-cancer cells. Concerning bitter acids, the tendency seems reversed; however, the number of studies is so limited for each time point that no robust conclusions can be drawn.

Next, the IC_50_ values of hop crude extracts were also estimated via meta-analysis. As shown in Table 4, the crude extracts exerted higher antiproliferative activity against cancer compared to non-cancer cells after 72 h of incubation (35.23 μg/mL, compared to 43.08 μg/mL). It should also be mentioned herein that the extracts were either hydroalcoholic (EtOH: H_2_O 9:1 *v*/*v*) or CO_2_-based supercritical fluid extracts (SFE).

A key characteristic of a potential anticancer agent is its ability to selectively present antiproliferative effects on cancer cell lines while sparing non-cancer cells. To evaluate this property, a meta-regression analysis was conducted. As presented in Table 5, only chalcones demonstrated statistically significant differences in IC_50_ values (*p*-values < 0.05) between cancer and non-cancer cells at all time points (24, 48 and 72 h). In contrast, no statistically significant differences were observed for flavones and beta acids. However, it is important to note that the number of studies available for the last two categories was considerably lower than that for chalcones, implying that at least these results should be interpreted with caution.

## 3. Discussion

Natural products, especially plant-derived compounds, have been crucial in drug discovery, particularly for cancer and infectious diseases. Over 60% of cancer drugs and 75% of treatments for infectious disease originate from natural sources. Nearly 50% of prescribed drugs in Europe and the USA are obtained from natural products or their derivatives [80]. Despite an estimated 250,000 to 500,000 plant species, only 1–10% have been extensively studied for medicinal use [81]. Interest in plant-based cancer treatments is rising due to their potential for lower toxicity compared to conventional therapies. *Humulus lupulus*, long used in beer production, presents a promising, low-toxicity alternative for anticancer drug development [82]. The cancer chemo-preventive activity of hop compounds was first reported as early as 1999 [3,79]. Since then, a variety of assays have been employed, including MTT and other tetrazolium salt-based assays, SRB and CV, each based on distinct chemical principles that target different cellular components involved in metabolic or structural processes [83,84,85]. Although of absolute need, there is no unique, straightforward and universally accepted method that can be used as the gold standard to evaluate anticancer potential of a compound or plant extract [86,87]. Given the growing body of evidence on the antiproliferative effects of various phytochemicals, including hop constituents, pharmaceutical companies and healthcare professionals increasingly demand a reliable index to quantify and compare the efficacy of these plant-derived compounds and extracts. When methodological comparisons yield conflicting results or hinder the ability to draw reliable conclusions, statistical approaches, particularly meta-analysis, have been recruited to provide an alternative perspective to resolve discrepancies and enhance clarity [25,88,89,90].

Meta-analysis enables the synthesis of findings from numerous studies, even in the presence of heterogeneity, thereby allowing for broader generalizations of an effect [91]. By quantitatively integrating data from diverse methodological approaches on a given topic, it helps identify the most reliable practices that may serve as potential gold standards [92]. Additionally, meta-analysis can reveal sources of variability in outcomes, providing deeper insights into the overall phenomenon and highlighting factors that influence the observed results [93]. This meta-analysis is the first attempt to quantitatively synthesize all available published evidence and evaluate the antiproliferative effects of several hop phytoconstituents across a range of cancerous and non-cancerous cell lines. Rather than evaluating the advantages or limitations of specific assay techniques, the analysis focuses on comparing antiproliferative activity across multiple incubation time points and assay types. The primary goal is to identify factors contributing to variability in test outcomes, reduce methodological inconsistencies, determine assay equivalency in terms of comparable outcomes and synthesize data to lend strength to their conclusions.

Given that half of our retrieved data involved XN, we took this opportunity to examine whether variations in antiproliferative assays results were influenced by inherent methodological variations. Our meta-regression-based approach demonstrated that data stratified by incubation time could be validly combined into a single meta-analysis. Furthermore, despite the limited number of studies available for two additional hop compounds—isoxanthohumol (IXN) and 8-prenylnaringenin (8-PN)—we employed the standardized mean difference (SMD) method to confirm that results across different assay types, including tetrazolium salt-based, SRB and crystal violet assays, are comparable and can be used interchangeably.

Our results further demonstrate that XN exerts a strong antiproliferative effect against most cancer cell lines, while showing significantly weaker activity on normal cell lines. These findings align with those of Viegas et al. [13], who demonstrated that XN (and beer containing hops) can mitigate the mutagenic effects of MeIQx (2-amino-3,8 dimethylimidazo[4,5-f]quinoxaline) and PhIP (2-amino-1-methyl-6-phenylimidazo[4,5-b]pyridine), two prevalent heterocyclic aromatic amines found in grilled meat. This protective effect was demonstrated in both the Salmonella typhimurium TA98 and in rat models, where it reduced the formation of aberrant crypt foci in the colon [94]. The fact that the antiproliferative activity of XN is highly dependent on the type of cancer cell line likely reflects the diverse mechanisms through which this activity is exerted. This notion is supported by numerous studies providing evidence that the anticancer properties of XN involve multiple pathways, many of which remain only partially understood. Proposed mechanisms include inhibition of cancer cell proliferation and migration, suppression of angiogenesis, induction of apoptosis or autophagy and cell cycle arrest [16,40,68,73,79,95,96,97,98]. In chronic myelogenous leukemia (CML), XN has also been shown to degrade the BCR-ABL fusion oncoprotein through caspase-mediated apoptosis [15], as well as via MAPK-related signaling pathways, including ERK and JNK [16,99]. In addition, despite the variation in IC_50_ values for the antiproliferative effect of XN on various cancer cell lines, the effect was clearly dependent on the incubation time. This time-dependent effect was consistently observed across all hop compounds tested and in both cancerous and non-cancerous cell lines.

A particularly intriguing finding of this meta-analysis is the notably strong antiproliferative activity of hop bitter acids—an aspect that has been largely overlooked and remains scarcely studied. Our results show that, among flavonoids, chalcones and particularly XN exhibit greater antiproliferative activity compared to flavones. Intriguingly, bitter acids (especially the beta acid, lupulone) demonstrated even more potent antiproliferative activity. Although beta acids also showed high activity against normal cells, this finding opens new avenues for the potential medicinal application of hops. However, given that the results on normal cell lines are based on only two studies, this outcome should be interpreted with caution. Since more than 50% of an SFE hop extract consists of alpha and beta acids, it is plausible that antiproliferative effects of hops extracts are highly shaped by bitter acids [12]. In line with this, our results showed that crude hop extracts exhibited strong antiproliferative activity, with greater potency against cancerous than non-cancerous cells.

This meta-analysis is subject to several limitations inherent in the individual studies, which may have affected the integrity of our results. The studies included encompassed a wide array of hop phytoconstituents, either purchased, isolated from hops or hop-spent extracts or chemically synthesized from extracts. However, the lack of information on the extraction procedures, compound purity levels, sources and detailed chemical characteristics may have introduced confounding factors that could not be adequately addressed in our analysis. Additional sources of variability include differences in compound dosing regimens, the use of various solvents for dilution (e.g., DMSO, EtOH, MeOH, SFE), inconsistencies in cell handling protocols, the timing of result acquisition and the subjective interpretation of outcomes. These factors are often underreported or inconsistently documented in the literature, adding further complexity to the meta-analysis. Moreover, the lack of standardized reporting regarding the origin, ATCC codes or classification of the cell lines used raises the risk of cell line misidentification and contributes to uncertainty in our pooled estimates, increasing between-study heterogeneity. A high degree of heterogeneity was expected and indeed observed in most of our analyses, likely reflecting inconsistencies in study design. Nonetheless, we applied a random-effects model in this meta-analysis, which assumes that variability is inherent to the biological questions being investigated [100]. Finally, despite the authors’ extensive efforts to systematically include all relevant studies—spanning conference proceedings, theses and publications in multiple languages—we cannot fully rule out the possibility of “gray literature bias” [101].

## 4. Materials and Methods

### 4.1. Literature Search Strategy

The Preferred Reporting Items for Systematic reviews and Meta-Analyses (PRISMA) guidelines [102] and the advice for best practices were followed to conduct this systematic review and meta-analysis [103]. The literature search was conducted in PubMed with the following search terms: (hop OR hops OR “*Humulus lupulus*”) AND (cancer OR *carcinoma OR neoplasm OR tumor OR proliferation) and all possible chemical compounds of hops [17], their synonyms and combinations, by 1 June 2024. The reference lists of selected articles were also scrutinized. Five researchers (GT, EMA, PP, KT and PK) independently evaluated search results, and disagreements in the initial evaluation were resolved after discussion with three separate reviewers (MEG, PB and GB).

### 4.2. Study Selection Criteria

For a study to be eligible for the present meta-analysis, it had to meet the following criteria: (i) it should evaluate the effects of hop compounds or hop extracts on the viability of cancer or non-cancer cells and no additional anticancer drugs or agents should be used; and (ii) it should provide IC_50_ values along with their corresponding standard deviation (SD) or standard error of the mean (SEM), or the necessary data that allow the calculation of them [104]. No language restrictions were applied in order to minimize the risk of publication bias associated with gray literature [105].

The assays used for assessing inhibition of cell growth in all studies included in this meta-analysis were sulforhodamine B (SRB), tetrazolium salt-based and crystal violet (CV) assays. MTT assay is classified among methods measuring metabolic activity and is a tetrazolium salt-based method, where a yellow tetrazolium salt (3-(4,5-dimethylthiazol-2-yl)-2,5-diphenyltetrazolium bromide or MTT) is reduced to purple formazan crystals by metabolically active cells, thus making it a fast and accurate method to measure the number of living cells [22]. The reaction taking place reflects the number of viable cells (as a cellular redox state) depending on mitochondrial redox enzyme activity [106]. Modifications of the above assay, including XTT (2,3-bis-(2-methoxy-4-nitro-5-sulfophenyl)-2H-tetrazolium-5-carboxanilide), MTS (3-(4,5-dimethylthiazol-2-yl)-5-(3-carboxymethoxyphenyl)-2-(4-sulfophenyl)-2H-tetrazolium) and WST (water-soluble tetrazolium salt), involve the replacement of MTT solution with other tetrazolium salts [107,108,109]. The sulforhodamine B (SRB) method is based on the ability of the chemical compound SRB to bind, under mild acidic conditions, to the amino acids of cellular proteins. Colorimetric evaluation provides information on the total mass of the proteins, which is directly correlated with the number of cells [21,110]. Crystal violet (CV) method is used for assessing the action of various chemotherapeutics and other agents on the growth and survival of a cell culture. It is based on the ability of living cells to remain attached to the plate. During cell death, the cells detach and can be easily removed from the living population throughout the assay. CV binds to the DNA of the cells, thus revealing a characteristic intense purple color proportional to the leaving cells [23].

### 4.3. Studies’ Outcomes and Data Extraction

Primarily, the titles and abstracts of the articles were screened, and relevant articles were further evaluated based on the inclusion and exclusion criteria. The search results were reviewed independently by three researchers (GT, EMA and PK). Any discrepancies were resolved through discussion with GS, PB and GB, and decisions were made by consensus. Experiments used many compounds found in hop (hop cones) extracts, which were tested at many different time points (mainly for 24, 48 and 72 h of incubation). Antiproliferative activity was investigated in many different cell lines. Data extracted from each study, and recorded on a spreadsheet, included the following: PubMed ID, first author’s last name, year of publication, type of assay for the determination of cell viability, cell lines, cancer type and hours of incubation and number of replicative experiments. In addition, the different compounds used to detect their antiproliferative activity as well as their corresponding IC_50_ values along with standard deviation or standard error of means (SD/SEM) were also recorded [111]. For studies reporting only the SD value, the number of replicates were used to calculate the SEM in the following way: SEM = SD/√n. Because studies lacking a reported SD still contribute meaningful information, excluding them could bias the pooled effect. Furukawa et al. [112] showed that SDs for the same outcome rarely differ significantly and suggested imputing a single pooled SD when necessary. To be even more conservative, we imputed any missing dispersion by using the largest SD observed among studies evaluating the same pair (compound–cell line), thereby intentionally down-weighting those studies while still preserving their contribution to the meta-analysis. We also included studies that reported data for both cancer and non-cancer cells.

### 4.4. Data Analysis

IC_50_ was used as the effect size of choice to test the antiproliferative effect of each compound. Data were combined using random-effects meta-analysis [113] with inverse variance. IC_50_ values were calculated along with their 95% confidence intervals (CIs) for each compound, in each type of cancer, at each incubation time and for each cell viability assay. Meta-regression analysis was applied to investigate the probability of statistical heterogeneity in terms of study-level variables between variances such as time of incubation or type of assay [114]. The analysis was performed using Stata 13 [115] by using the commands “metan” and “metareg” for random-effect meta-analysis [116] and meta-regression with method of moments [117], respectively. For testing statistical significance, *p* < 0.05 was used as the decision rule, and meta-analysis was performed where two or more studies were available.

## 5. Conclusions

In conclusion, this meta-analysis provides valuable insights into the antiproliferative effects of hop compounds, particularly xanthohumol (XN), which demonstrated strong activity against cancer cell lines while sparing normal cells. Our findings underscore the therapeutic potential of hop-derived phytochemicals, including bitter acids like humulone and lupulone, in cancer treatment. However, further research—including preclinical and clinical studies—is necessary to validate these effects. Despite the inherent limitations and variability in study designs contributing to heterogeneity in some subgroup analyses [118], this analysis offers a comprehensive synthesis of available data and highlights the urgent need for standardized methodologies to evaluate the anticancer potential of plant-derived compounds. Importantly, our results indicate that tetrazolium salt-based assays, SRB and crystal violet (CV) assays yield comparable outcomes and can be used interchangeably in studies involving hop compounds. The methodological framework applied in this study may also be extended to other plant extracts and phytoconstituents, facilitating the identification of distinct bioactivities and advancing our understanding of the health-promoting properties of medicinal plants.

## Figures and Tables

**Table 1:** Characteristics of the 61 studies included in the meta-analysis.

Author Reference	Assay	IC_50_ (nM or mg/mL for Extracts)	SD	Hours	Number of Replicates	Cell Line	Cell Type	Cancer/Normal	Compound or Type of Extract
Hsieh et al. [16]	MTT	56.00	7.30	24	3	NPC-039	Human neck cancer	Neck	Xanthohumol
Hsieh et al. [16]	MTT	56.00	7.30	24	3	NPC-039	Human neck cancer	Neck	Xanthohumol
Hsieh et al. [16]	MTT	45.60	7.30	24	3	NPC-BM	Human neck cancer	Neck	Xanthohumol
Hsieh et al. [16]	MTT	34.90	6.00	24	3		Human normal nasopharyngeal	Non-cancer	Xanthohumol
Hsieh et al. [16]	MTT	22.80	7.30	48	3	NPC-039	Human neck cancer	Neck	Xanthohumol
Hsieh et al. [16]	MTT	18.10	7.30	48	3	NPC-BM	Human neck cancer	Neck	Xanthohumol
Hsieh et al. [16]	MTT	20.00	6.00	48	3		Human normal nasopharyngeal	Non-cancer	Xanthohumol
Hsieh et al. [16]	MTT	19.50	7.30	72	3	NPC-039	Human neck cancer	Neck	Xanthohumol
Hsieh et al. [16]	MTT	17.80	7.30	72	3	NPC-BM	Human neck cancer	Neck	Xanthohumol
Hsieh et al. [16]	MTT	20.00	6.00	72	3		Human normal nasopharyngeal	Non-cancer	Xanthohumol
Klimek et al. [12]	MTT	74.94	2.62	24	3	BJ	Human normal skin fibroblast	Non-cancer	Xanthohumol
Klimek et al. [12]	MTT	34.36	2.89	72	3	MCF-7	Human breast cancer	Breast	Xanthohumol
Klimek et al. [12]	MTT	20.85	2.99	72	3	A549	Human lung cancer	Lung	Xanthohumol
Klimek et al. [12]	MTT	102.59	3.48	72	3	HepG2	Human liver cancer	Liver	Xanthohumol
Klimek et al. [12]	MTT	48.67	1.35	72	3	BJ	Human normal skin fibroblast	Non-cancer	Xanthohumol
Klimek et al. [12]	MTT	155.70	4.23	24	3	BJ	Human normal skin fibroblast	Non-cancer	Hops dynamic supercritical fluid extract (SFE)
Klimek et al. [12]	MTT	45.17	3.58	72	3	A549	Human lung cancer	Lung	Hops dynamic supercritical fluid extract (SFE)
Klimek et al. [12]	MTT	66.48	2.97	72	3	MCF-7	Human breast cancer	Breast	Hops dynamic supercritical fluid extract (SFE)
Klimek et al. [12]	MTT	26.27	1.56	72	3	HepG2	Human liver cancer	Liver	Hops dynamic supercritical fluid extract (SFE)
Klimek et al. [12]	MTT	104.30	4.16	72	3	BJ	Human normal skin fibroblast	Non-cancer	Hops dynamic supercritical fluid extract (SFE)
Hitzman et al. [4]	MTT	76.00	6.91	24	3	MCF-7	Human breast cancer	Breast	Xanthohumol
Hitzman et al. [4]	MTT	32.80	0.40	24	3	MCF-7	Human breast cancer	Breast	Spent hops ethanolic extract, LC-MS characterized (33.20% XH, 1.22% 6-PN, 1.11% isoxanthohumol and 0.28% 8-PN)
Hitzman et al. [4]	MTT	105.00	6.91	24	3	MCF-7	Human breast cancer	Breast	6-prenylnaringenin
Hitzman et al. [4]	MTT	115.00	8.20	24	3	MCF-7	Human breast cancer	Breast	8-prenylnaringenin
Yin et al. [27]	MTT	7.90	7.30	24	3	KYSE30	Human esophageal cancer	Esophagus	Xanthohumol
Yin et al. [27]	MTT	3.10	7.30	48	3	KYSE30	Human esophageal cancer	Esophagus	Xanthohumol
Yin et al. [27]	MTT	2.60	7.30	72	3	KYSE30	Human esophageal cancer	Esophagus	Xanthohumol
Ho et al. [28]	MTT	60.00	7.30	24	3	U87-MG	Human glioblastoma	Glioblastoma	Xanthohumol
Ho et al. [28]	MTT	68.10	7.30	24	3	A172	Human glioblastoma	Glioblastoma	Xanthohumol
Scagliarini et al. [29]	Crystal Violet	39.00	6.48	24	3	HT-29	Human colon cancer	Colon	Xanthohumol
Scagliarini et al. [29]	Crystal Violet	12.00	2.8	48	3	HT-29	Human colon cancer	Colon	Xanthohumol
Scagliarini et al. [29]	Crystal Violet	22.00	6.49	48	3	SW480	Human colon cancer	Colon	Xanthohumol
Scagliarini et al. [29]	Crystal Violet	12.00	3.57	48	3	SW620	Human colon cancer	Colon	Xanthohumol
Scagliarini et al. [29]	Crystal Violet	10.00	1.75	72	3	HT-29	Human colon cancer	Colon	Xanthohumol
Scagliarini et al. [29]	Crystal Violet	20.00	3.3	72	3	SW480	Human colon cancer	Colon	Xanthohumol
Scagliarini et al. [29]	Crystal Violet	7.00	1.38	72	3	SW620	Human colon cancer	Colon	Xanthohumol
Stompor et al. [30]	SRB	8.8	1.16	72	4	MCF-7	Human breast cancer	Breast	Xanthohumol
Stompor et al. [30]	SRB	18.9	4.6	72	4	MDA-MB-231	Human breast cancer	Breast	Xanthohumol
Stompor et al. [30]	SRB	8.7	1.3	72	4	4T1	Murine breast cancer	Breast	Xanthohumol
Stompor et al. [30]	SRB	21.5	2.7	72	4	HepG2	Human liver cancer	Liver	Xanthohumol
Stompor et al. [30]	SRB	12.6	8.8	72	4	BALB/3T3	Murine normal fibroblasts	Non-cancer	Xanthohumol
Stompor et al. [30]	SRB	21.1	4.3	72	4	MCF-10A	Human normal breast	Non-cancer	Xanthohumol
Lu et al. [15]	MTT	39.82	1.50	24	3	K562	Human leukemia	Leukemia	Xanthohumol
Lu et al. [15]	MTT	19.56	0.77	48	3	K562	Human leukemia	Leukemia	Xanthohumol
Lu et al. [15]	MTT	4.43	1.20	72	3	K562	Human leukemia	Leukemia	Xanthohumol
Lu et al. [15]	MTT	10.00	1.50	72	3	K562/ADR	Human leukemia, adriamycin-resistant	Leukemia	Xanthohumol
Sławińska-Brych et al. [31]	MTT	50.12	7.30	48	3	U266	Human myeloma	Myeloma	Xanthohumol
Sławińska-Brych et al. [31]	MTT	21.85	7.30	48	3	RPMI8226	Human myeloma	Myeloma	Xanthohumol
Sławińska-Brych et al. [31]	MTT	38.40	7.30	96	3	U266	Human myeloma	Myeloma	Xanthohumol
Sławińska-Brych et al. [31]	MTT	8.24	7.30	96	3	RPMI8226	Human myeloma	Myeloma	Xanthohumol
Koosha et al. [32]	MTT	166.68	8.20	24	3	HCT116	Human colon cancer	Colon	8-prenylnaringenin
Koosha et al. [32]	MTT	70.00	2.90	48	3	HCT116	Human colon cancer	Colon	8-prenylnaringenin
Koosha et al. [32]	MTT	58.49	4.10	72	3	HCT116	Human colon cancer	Colon	8-prenylnaringenin
Krajnović et al. [33]	Crystal violet	42.00	5.19	48	3	B16-F10	Murine melanoma	Melanoma	Isoxanthohumol
Krajnović et al. [33]	MTT	30.00	4.05	48	3	B16-F10	Murine melanoma	Melanoma	Isoxanthohumol
Bocquet et al. [17]	MTT	7.10	0.80	48	3	HepG2	Human liver cancer	Liver	Xanthohumol
Bocquet et al. [17]	MTT	29.40	2.60	48	3	MG-63	Human osteosarcoma	Bone	Xanthohumol
Bocquet et al. [17]	MTT	19.50	0.50	48	3	WI-38	Human normal lung fibroblast	Non-cancer	Xanthohumol
Bocquet et al. [17]	MTT	9.60	0.50	48	3	J774	Mouse monocyte macrophage	Non-cancer	Xanthohumol
Bocquet et al. [17]	MTT	31.40	8.10	72	3	MG-63	Human osteosarcoma	Bone	Hydro-alcoholic extract: ethanol/water (9:1; *v*/*v*) 3 successive macerations of 4 h and 1 o/n, stirring in the dark
Bocquet et al. [17]	MTT	6.80	2.50	72	3	HepG2	Human liver cancer	Liver	Hydro-alcoholic extract: ethanol/water (9:1; *v*/*v*) 3 successive macerations of 4 h and 1 o/n, stirring in the dark
Bocquet et al. [17]	MTT	7.60	0.10	72	3	WI-38	Human normal lung fibroblast	Non-cancer	Hydro-alcoholic extract: ethanol/water (9:1; *v*/*v*) 3 successive macerations of 4 h and 1 o/n, stirring in the dark
Bocquet et al. [17]	MTT	19.70	2.80	72	3	J774	Mouse monocyte macrophage	Non-cancer	Hydro-alcoholic extract: ethanol/water (9:1; *v*/*v*) 3 successive macerations of 4 h and 1 o/n, stirring in the dark
Bocquet et al. [17]	MTT	2.60	0.10	48	3	WI-38	Human normal lung fibroblast	Non-cancer	Lupulone
Bocquet et al. [17]	MTT	3.60	0.10	48	3	J774	Mouse monocyte macrophage	Non-cancer	Lupulone
Bocquet et al. [17]	MTT	2.90	0.50	48	3	HepG2	Human liver cancer	Liver	Lupulone
Bocquet et al. [17]	MTT	10.40	0.40	48	3	MG-63	Human osteosarcoma	Bone	Lupulone
Bocquet et al. [17]	MTT	29.00	2.30	48	3	WI-38	Human normal lung fibroblast	Non-cancer	Humulone
Bocquet et al. [17]	MTT	31.70	0.30	48	3	J774	Mouse monocyte macrophage	Non-cancer	Humulone
Bocquet et al. [17]	MTT	178.50	2.50	48	3	WI-38	Human normal lung fibroblast	Non-cancer	Desmethylxanthohumol
Bocquet et al. [17]	MTT	28.50	1.00	48	3	J774	Mouse monocyte macrophage	Non-cancer	Desmethylxanthohumol
Bocquet et al. [17]	MTT	65.90	2.90	48	3	HepG2	Human liver cancer	Liver	Desmethylxanthohumol
Bocquet et al. [17]	MTT	116.20	3.30	48	3	MG-63	Human osteosarcoma	Bone	Desmethylxanthohumol
Roehrer et al. [34]	MTS	12.25	6.91	48	3	MCF-7	Human breast cancer	Breast	Xanthohumol
Roehrer et al. [34]	MTS	8.80	7.30	96	3	MCF-7	Human breast cancer	Breast	Xanthohumol
Logan et al. [11]	SRB	40.8	1.4	24	5	HCT116	Human colon cancer	Colon	Xanthohumol
Logan et al. [11]	SRB	50.2	1.4	24	5	HT-29	Human colon cancer	Colon	Xanthohumol
Logan et al. [11]	SRB	25.4	1.1	24	5	HepG2	Human liver cancer	Liver	Xanthohumol
Logan et al. [11]	SRB	37.2	1.5	24	5	Huh7	Human liver cancer	Liver	Xanthohumol
Bartmańska et al. [35]	SRB	10.84	0.32	72	5	MCF-7	Human breast cancer	Breast	Xanthohumol
Bartmańska et al. [35]	SRB	8.46	3.19	72	5	MDA-MB-231	Human breast cancer	Breast	Xanthohumol
Bartmańska et al. [35]	SRB	7.99	2.77	72	5	T-47D	Human breast cancer	Breast	Xanthohumol
Bartmańska et al. [35]	SRB	9.42	0.25	72	5	HT-29	Human colon cancer	Colon	Xanthohumol
Bartmańska et al. [35]	SRB	2.06	1.03	72	5	A2780	Human ovarian cancer	Ovarian	Xanthohumol
Bartmańska et al. [35]	SRB	8.21	0.83	72	5	A2780	Human ovarian cancer	Ovarian	Xanthohumol
Bartmańska et al. [35]	SRB	6.49	2.14	72	5	DU145	Human prostate cancer	Prostate	Xanthohumol
Bartmańska et al. [35]	SRB	8.61	1.11	72	5	PC-3	Human prostate cancer	Prostate	Xanthohumol
Bartmańska et al. [35]	SRB	9.57	4.23	72	5	HLMEC	Human lung microvascular endothelial	Non-cancer	Xanthohumol
Bartmańska et al. [35]	SRB	55.95	27.31	72	5	MCF-10A	Human normal breast	Non-cancer	Xanthohumol
Bartmańska et al. [35]	SRB	43.25	4.37	72	5	MCF-7	Human breast cancer	Breast	6-prenylnaringenin
Bartmańska et al. [35]	SRB	62.64	19.54	72	5	MDA-MB-231	Human breast cancer	Breast	6-prenylnaringenin
Bartmańska et al. [35]	SRB	16.01	3.74	72	5	T-47D	Human breast cancer	Breast	6-prenylnaringenin
Bartmańska et al. [35]	SRB	64.61	17.07	72	5	HT-29	Human colon cancer	Colon	6-prenylnaringenin
Bartmańska et al. [35]	SRB	44.16	14.71	72	5	A2780	Human ovarian cancer	Ovarian	6-prenylnaringenin
Bartmańska et al. [35]	SRB	81.73	17.68	72	5	A2780	Human ovarian cancer	Ovarian	6-prenylnaringenin
Bartmańska et al. [35]	SRB	79.56	8.89	72	5	DU145	Human prostate cancer	Prostate	6-prenylnaringenin
Bartmańska et al. [35]	SRB	75.53	29.79	72	5	PC-3	Human prostate cancer	Prostate	6-prenylnaringenin
Bartmańska et al. [35]	SRB	13.69	5.16	72	5	HLMEC	Human lung microvascular endothelial	Non-cancer	6-prenylnaringenin
Bartmańska et al. [35]	SRB	110.06	32.95	72	5	MCF-10A	Human normal breast	Non-cancer	6-prenylnaringenin
Bartmańska et al. [35]	SRB	49.53	7.36	72	5	MCF-7	Human breast cancer	Breast	8-prenylnaringenin
Bartmańska et al. [35]	SRB	63.81	7.27	72	5	MDA-MB-231	Human breast cancer	Breast	8-prenylnaringenin
Bartmańska et al. [35]	SRB	26.71	9.7	72	5	T-47D	Human breast cancer	Breast	8-prenylnaringenin
Bartmańska et al. [35]	SRB	89.84	3.42	72	5	HT-29	Human colon cancer	Colon	8-prenylnaringenin
Bartmańska et al. [35]	SRB	25.91	8.32	72	5	A2780	Human ovarian cancer	Ovarian	8-prenylnaringenin
Bartmańska et al. [35]	SRB	66.37	10.14	72	5	A2780	Human ovarian cancer	Ovarian	8-prenylnaringenin
Bartmańska et al. [35]	SRB	60.58	6.66	72	5	DU145	Human prostate cancer	Prostate	8-prenylnaringenin
Bartmańska et al. [35]	SRB	51.36	11.31	72	5	PC-3	Human prostate cancer	Prostate	8-prenylnaringenin
Bartmańska et al. [35]	SRB	23.91	10.86	72	5	HLMEC	Human lung microvascular endothelial	Non-cancer	8-prenylnaringenin
Bartmańska et al. [35]	SRB	90.72	19.8	72	5	MCF-10A	Human normal breast	Non-cancer	8-prenylnaringenin
Bartmańska et al. [35]	SRB	16.73	0.88	72	5	MCF-7	Human breast cancer	Breast	Isoxanthohumol
Bartmańska et al. [35]	SRB	43.34	10.32	72	5	MDA-MB-231	Human breast cancer	Breast	Isoxanthohumol
Bartmańska et al. [35]	SRB	26.75	6.44	72	5	T-47D	Human breast cancer	Breast	Isoxanthohumol
Bartmańska et al. [35]	SRB	30.59	1	72	5	HT-29	Human colon cancer	Colon	Isoxanthohumol
Bartmańska et al. [35]	SRB	7.93	1.65	72	5	A2780	Human ovarian cancer	Ovarian	Isoxanthohumol
Bartmańska et al. [35]	SRB	11.65	1.44	72	5	A2780	Human ovarian cancer	Ovarian	Isoxanthohumol
Bartmańska et al. [35]	SRB	59.17	5.73	72	5	DU145	Human prostate cancer	Prostate	Isoxanthohumol
Bartmańska et al. [35]	SRB	53.24	10.59	72	5	PC-3	Human prostate cancer	Prostate	Isoxanthohumol
Bartmańska et al. [35]	SRB	12.5	5.65	72	5	HLMEC	Human lung microvascular endothelial	Non-cancer	Isoxanthohumol
Bartmańska et al. [35]	SRB	72.12	21.66	72	5	MCF-10A	Human normal breast	Non-cancer	Isoxanthohumol
Bartmańska et al. [35]	SRB	130.79	6.11	72	5	MCF-7	Human breast cancer	Breast	Naringenin
Bartmańska et al. [35]	SRB	166.09	82.44	72	5	MDA-MB-231	Human breast cancer	Breast	Naringenin
Bartmańska et al. [35]	SRB	104.53	48.31	72	5	T-47D	Human breast cancer	Breast	Naringenin
Bartmańska et al. [35]	SRB	130.8	28.19	72	5	HT-29	Human colon cancer	Colon	Naringenin
Bartmańska et al. [35]	SRB	100.05	4.77	72	5	A2780	Human ovarian cancer	Ovarian	Naringenin
Bartmańska et al. [35]	SRB	109.23	16.98	72	5	A2780	Human ovarian cancer	Ovarian	Naringenin
Bartmańska et al. [35]	SRB	133.66	12.92	72	5	DU145	Human prostate cancer	Prostate	Naringenin
Bartmańska et al. [35]	SRB	171.23	28.78	72	5	PC-3	Human prostate cancer	Prostate	Naringenin
Bartmańska et al. [35]	SRB	117.24	32.27	72	5	HLMEC	Human lung microvascular endothelial	Non-cancer	Naringenin
Bartmańska et al. [35]	SRB	187.1	72.41	72	5	MCF-10A	Human normal breast	Non-cancer	Naringenin
Bartmańska et al. [35]	SRB	10.07	2.31	72	5	MCF-7	Human breast cancer	Breast	α,β-dihydroxanthohumol
Bartmańska et al. [35]	SRB	10.02	3.26	72	5	MDA-MB-231	Human breast cancer	Breast	α,β-dihydroxanthohumol
Bartmańska et al. [35]	SRB	7.27	3.05	72	5	T-47D	Human breast cancer	Breast	α,β-dihydroxanthohumol
Bartmańska et al. [35]	SRB	12.23	2.99	72	5	HT-29	Human colon cancer	Colon	α,β-dihydroxanthohumol
Bartmańska et al. [35]	SRB	1.8	0.64	72	5	A2780	Human ovarian cancer	Ovarian	α,β-dihydroxanthohumol
Bartmańska et al. [35]	SRB	11.59	3.36	72	5	A2780	Human ovarian cancer	Ovarian	α,β-dihydroxanthohumol
Bartmańska et al. [35]	SRB	12.96	4.2	72	5	DU145	Human prostate cancer	Prostate	α,β-dihydroxanthohumol
Bartmańska et al. [35]	SRB	16.27	5.22	72	5	PC-3	Human prostate cancer	Prostate	α,β-dihydroxanthohumol
Bartmańska et al. [35]	SRB	14.17	4.24	72	5	HLMEC	Human lung microvascular endothelial	Non-cancer	α,β-dihydroxanthohumol
Bartmańska et al. [35]	SRB	72.05	8.55	72	5	MCF-10A	Human normal breast	Non-cancer	α,β-dihydroxanthohumol
Wei et al. [36]	MTS	16.04	7.30	24	3	AGS	Human gastric cancer	Gastric	Xanthohumol
Wei et al. [36]	MTS	111.16	7.30	24	3	SGC-7901	Human gastric cancer	Gastric	Xanthohumol
Wei et al. [36]	MTS	35.81	7.30	24	3	MGC-803	Human gastric cancer	Gastric	Xanthohumol
Wei et al. [36]	EdU	8.00	7.30	24	3	AGS	Human gastric cancer	Gastric	Xanthohumol
Wei et al. [36]	MTS	285.26	6.00	24	3	GES-1	Human normal gastric	Non-cancer	Xanthohumol
Carvalho et al. [37]	SRB	85.5	8.92	24	3	TPC-1	Human thyroid cancer	Thyroid	Xanthohumol
Carvalho et al. [37]	SRB	59	8.92	48	3	TPC-1	Human thyroid cancer	Thyroid	Xanthohumol
Carvalho et al. [37]	SRB	48.5	8.92	72	3	TPC-1	Human thyroid cancer	Thyroid	Xanthohumol
Ho et al. [38]	MTT	53.70	7.30	24	3	M059K	Human glioblastoma	Glioblastoma	Xanthohumol
Ho et al. [38]	MTT	55.60	7.30	24	3	U87-MG	Human glioblastoma	Glioblastoma	Xanthohumol
Popłoński et al. [39]	SRB	8.1	0.8	72	4	MCF-7	Human breast cancer	Breast	Xanthohumol
Popłoński et al. [39]	SRB	10.1	1.1	72	4	HT-29	Human colon cancer	Colon	Xanthohumol
Popłoński et al. [39]	SRB	7	1.5	72	4	PC-3	Human prostate cancer	Prostate	Xanthohumol
Sun et al. [40]	MTT	39.40	6.91	24	6	MCF-7	Human breast cancer	Breast	Xanthohumol
Sun et al. [40]	MTT	33.30	7.30	24	6	MDA-MB-231	Human breast cancer	Breast	Xanthohumol
Sun et al. [40]	MTT	19.60	6.91	48	6	MCF-7	Human breast cancer	Breast	Xanthohumol
Sun et al. [40]	MTT	21.50	7.30	48	6	MDA-MB-231	Human breast cancer	Breast	Xanthohumol
Sun et al. [40]	MTT	61.10	6.00	48	6	h-TERT-BJ	Human normal skin fibroblast	Non-cancer	Xanthohumol
Sun et al. [40]	MTT	135.30	6.00	48	6	MCF-10A	Human normal breast	Non-cancer	Xanthohumol
Saito et al. [41]	WST-1	17.00	7.30	72	6	BxPC-3	Human pancreatic cancer	Pancreas	Xanthohumol
Saito et al. [41]	WST-1	15.90	7.30	72	6	MIA PaCa-2	Human pancreatic cancer	Pancreas	Xanthohumol
Saito et al. [41]	WST-1	12.90	7.30	72	6	AsPC-1	Human pancreatic cancer	Pancreas	Xanthohumol
Stompor et al. [42]	SRB	30	3.8	72	3	MCF-7	Human breast cancer	Breast	Isoxanthohumol
Stompor et al. [42]	SRB	29.7	4.2	72	3	A549	Human lung cancer	Lung	Isoxanthohumol
Stompor et al. [42]	SRB	8.96	1.5	72	3	LoVo	Human colon cancer	Colon	Isoxanthohumol
Stompor et al. [42]	SRB	26.8	4	72	3	LoVo	Human colon cancer	Colon	Isoxanthohumol
Stompor et al. [42]	SRB	16	3.6	72	3	MES-SA	Human uterine cancer	Uterus	Isoxanthohumol
Stompor et al. [42]	SRB	30.4	4.1	72	3	MES-SA	Human uterine cancer	Uterus	Isoxanthohumol
Stompor et al. [42]	SRB	37.1	3.8	72	3	MCF-10A	Human normal breast	Non-cancer	Isoxanthohumol
Stompor et al. [42]	XTT	15.60	4.05	72	3	U-118 MG	Human glioblastoma	Glioblastoma	Isoxanthohumol
Liu et al. [43]	MTT	81.45	6.91	24	3	MCF-7	Human breast cancer	Breast	Xanthohumol
Liu et al. [43]	MTT	78.33	7.30	24	3	MCF-7/ADR	Human breast cancer, doxorubicin-resistant	Breast	Xanthohumol
Liu et al. [43]	MTT	34.02	3.45	48	3	MCF-7	Human breast cancer	Breast	Xanthohumol
Liu et al. [43]	MTT	33.71	3.12	48	3	MCF-7/ADR	Human breast cancer, doxorubicin-resistant	Breast	Xanthohumol
Liu et al. [43]	MTT	11.22	0.95	72	3	MCF-7	Human breast cancer	Breast	Xanthohumol
Liu et al. [43]	MTT	11.37	1.15	72	3	MCF-7/ADR	Human breast cancer, doxorubicin-resistant	Breast	Xanthohumol
Gallo et al. [44]	MTT	18.30	6.00	96	3	HUVEC	Human umbilical vein endothelial	Non-cancer	Xanthohumol
Chen et al. [45]	MTT	64.80	7.30	24	3	U87-MG	Human glioblastoma	Glioblastoma	Xanthohumol
Chen et al. [45]	MTT	19.70	7.30	48	3	U87-MG	Human glioblastoma	Glioblastoma	Xanthohumol
Chen et al. [45]	MTT	13.10	7.30	72	3	U87-MG	Human glioblastoma	Glioblastoma	Xanthohumol
Lempereur et al. [46]	Crystal Violet	4.10	11.60	72	6	MCF-7	Human breast cancer	Breast	Tetrahydro Iso-Alpha Acids
Lempereur et al. [46]	Crystal Violet	20.60	11.60	72	6	MDA-MB-231	Human breast cancer	Breast	Tetrahydro Iso-Alpha Acids
Lempereur et al. [46]	Crystal Violet	15.30	11.60	72	6	MCF-7	Human breast cancer	Breast	α-acids
Lempereur et al. [46]	Crystal Violet	15.70	11.60	72	6	MDA-MB-231	Human breast cancer	Breast	α-acids
Lempereur et al. [46]	Crystal Violet	13.10	11.60	72	6	MCF-7	Human breast cancer	Breast	Iso-α-acids
Lempereur et al. [46]	Crystal Violet	13.70	11.60	72	6	MDA-MB-231	Human breast cancer	Breast	Iso-α-acids
Lempereur et al. [46]	Crystal Violet	29.90	11.60	72	6	MDA-MB-231	Human breast cancer	Breast	Dihydro-iso-alpha acids
Yoo et al. [47]	MTT	16.80	7.30	48	4	MDA-MB-231	Human breast cancer	Breast	Xanthohumol
Krajnović et al. [48]	Crystal Violet	15.77	1.74	48	3	A375	Human melanoma	Melanoma	Xanthohumol
Krajnović et al. [48]	Crystal Violet	9.97	2.32	48	3	B16	Murine melanoma	Melanoma	Xanthohumol
Krajnović et al. [48]	Crystal Violet	48.30	11.6	48	3	A375	Human melanoma	Melanoma	8-prenylnaringenin
Krajnović et al. [48]	Crystal Violet	38.55	8.84	48	3	B16	Murine melanoma	Melanoma	8-prenylnaringenin
Krajnović et al. [48]	Crystal Violet	24.18	1.43	48	3	A375	Human melanoma	Melanoma	Isoxanthohumol
Krajnović et al. [48]	Crystal Violet	21.88	5.19	48	3	B16	Murine melanoma	Melanoma	Isoxanthohumol
Krajnović et al. [48]	MTT	15.00	1.15	48	3	A375	Human melanoma	Melanoma	Xanthohumol
Krajnović et al. [48]	MTT	8.70	0.99	48	3	B16	Murine melanoma	Melanoma	Xanthohumol
Krajnović et al. [48]	MTT	27.80	3.82	48	3	A375	Human melanoma	Melanoma	8-prenylnaringenin
Krajnović et al. [48]	MTT	40.85	0.78	48	3	B16	Murine melanoma	Melanoma	8-prenylnaringenin
Krajnović et al. [48]	MTT	22.90	0.78	48	3	A375	Human melanoma	Melanoma	Isoxanthohumol
Krajnović et al. [48]	MTT	22.15	4.05	48	3	B16	Murine melanoma	Melanoma	Isoxanthohumol
Yong et al. [49]	SRB	74.06	3.43	24	3	A549	Human lung cancer	Lung	Xanthohumol
Yong et al. [49]	SRB	25.48	0.52	48	3	A549	Human lung cancer	Lung	Xanthohumol
Yong et al. [49]	SRB	149.2	8.59	48	3	MRC-5	Human normal lung fibroblast	Non-cancer	Xanthohumol
Yong et al. [49]	SRB	13.5	1.42	72	3	A549	Human lung cancer	Lung	Xanthohumol
Yong et al. [49]	SRB	94.38	3.07	72	3	MRC-5	Human normal lung fibroblast	Non-cancer	Xanthohumol
Zhang et al. [50]	CCK-8	7.90	7.30	48	3	HeLa	Human cervical cancer	Cervix	Xanthohumol
Zhang et al. [50]	CCK-8	8.60	2.99	48	3	A549	Human lung cancer	Lung	Xanthohumol
Zhang et al. [50]	CCK-8	32.00	6.00	48	3	MCF-10A	Human normal breast	Non-cancer	Xanthohumol
Zhang et al. [50]	CCK-8	53.60	4.05	48	3	A549	Human lung cancer	Lung	Isoxanthohumol
Zhang et al. [50]	CCK-8	46.30	4.05	48	3	HeLa	Human cervical cancer	Cervix	Isoxanthohumol
Sławińska-Brych et al. [51]	MTT	12.30	7.30	48	3	RK33	Human neck cancer	Neck	Xanthohumol
Sławińska-Brych et al. [51]	MTT	22.50	7.30	48	3	RK45	Human neck cancer	Neck	Xanthohumol
Sławińska-Brych et al. [51]	MTT	100.00	6.00	48	3	HSF	Human normal skin fibroblast	Non-cancer	Xanthohumol
Sławińska-Brych et al. [51]	MTT	105.00	6.00	48	3	OLN93	Rat oligodendroglia	Non-cancer	Xanthohumol
Jiang et al. [52]	MTS	10.00	7.30	24	3	PANC-1	Human pancreatic cancer	Pancreas	Xanthohumol
Jiang et al. [52]	MTS	27.70	7.30	24	3	BxPC-3	Human pancreatic cancer	Pancreas	Xanthohumol
Jiang et al. [52]	MTS	8.10	7.30	48	3	PANC-1	Human pancreatic cancer	Pancreas	Xanthohumol
Jiang et al. [52]	MTS	9.10	7.30	48	3	BxPC-3	Human pancreatic cancer	Pancreas	Xanthohumol
Jiang et al. [52]	MTS	5.60	7.30	72	3	BxPC-3	Human pancreatic cancer	Pancreas	Xanthohumol
Jiang et al. [52]	MTS	4.40	7.30	72	3	PANC-1	Human pancreatic cancer	Pancreas	Xanthohumol
Zenger et al. [53]	MTT	65.00	4.00	24	3	HSC	Human hepatic stellate	Non-cancer	Xanthohumol
Yong and Abd Malek [54]	SRB	59.96	2.76	24	2	Ca Ski	Human cervical cancer	Cervix	Xanthohumol
Yong and Abd Malek [54]	SRB	34.01	1.6	48	2	Ca Ski	Human cervical cancer	Cervix	Xanthohumol
Yong and Abd Malek [54]	SRB	20.08	1.58	72	2	Ca Ski	Human cervical cancer	Cervix	Xanthohumol
Mouratidis et al. [55]	MTT	10.50	0.50	48	3	PC-3	Human prostate cancer	Prostate	Lupulone
Mouratidis et al. [55]	MTT	9.00	0.50	48	3	DU145	Human prostate cancer	Prostate	Lupulone
Mouratidis et al. [55]	MTT	5.00	0.50	72	3	PC-3	Human prostate cancer	Prostate	Lupulone
Mouratidis et al. [55]	MTT	5.00	0.50	72	3	DU145	Human prostate cancer	Prostate	Lupulone
Boncler et al. [56]	MTT	9.60	0.40	24	4	HUVEC	Human umbilical vein endothelial	Non-cancer	Spent hops, after SFE extraction of hops, were dried and then extracted with acetone:water (70:30; *v*/*v*)
Tronina et al. [57]	SRB	10.95	1.03	72	4	MCF-7	Human breast cancer	Breast	Xanthohumol
Tronina et al. [57]	SRB	91.31	8.92	72	4	HT-29	Human colon cancer	Colon	Xanthohumol
Tronina et al. [57]	SRB	10.67	1.06	72	4	PC-3	Human prostate cancer	Prostate	Xanthohumol
Tronina et al. [58]	SRB	10.95	1.03	72	4	MCF-7	Human breast cancer	Breast	Xanthohumol
Tronina et al. [58]	SRB	91.31	8.92	72	4	HT-29	Human colon cancer	Colon	Xanthohumol
Tronina et al. [58]	SRB	10.67	1.06	72	4	PC-3	Human prostate cancer	Prostate	Xanthohumol
Tronina et al. [58]	SRB	26.54	12.68	72	4	MCF-7	Human breast cancer	Breast	Isoxanthohumol
Tronina et al. [58]	SRB	88.82	4.15	72	4	HT-29	Human colon cancer	Colon	Isoxanthohumol
Tronina et al. [58]	SRB	71.32	19.59	72	4	PC-3	Human prostate cancer	Prostate	Isoxanthohumol
Tronina et al. [58]	SRB	9.15	0.62	72	4	MCF-7	Human breast cancer	Breast	α,β-dihydroxanthohumol
Tronina et al. [58]	SRB	74.41	23.44	72	4	HT-29	Human colon cancer	Colon	α,β-dihydroxanthohumol
Tronina et al. [58]	SRB	14.73	3.88	72	4	PC-3	Human prostate cancer	Prostate	α,β-dihydroxanthohumol
Allsopp et al. [59]	MTT	70.00	8.20	24	3	CaCo-2	Human colon cancer	Colon	8-prenylnaringenin
Allsopp et al. [59]	MTT	55.00	4.05	24	3	CaCo-2	Human colon cancer	Colon	Isoxanthohumol
Kang et al. [60]	MTT	16.60	6.91	24	3	MCF-7	Human breast cancer	Breast	Xanthohumol
Kang et al. [60]	MTT	18.00	7.30	24	3	MCF-7/ADR	Human breast cancer, doxorubicin-resistant	Breast	Xanthohumol
Kang et al. [60]	MTT	35.20	7.30	24	3	HT-29	Human colon cancer	Colon	Xanthohumol
Viegas et al. [13]	MTT	117.10	3.48	24	3	HepG2	Human liver cancer	Liver	Xanthohumol
Hemachandra et al. [61]	MTT	11.00	0.50	24	3	MCF-10A	Human normal breast	Non-cancer	Ethanolic extract: Spent hops, after SFE extraction of hops, were dried and then extracted with ethanol
Deeb et al. [62]	MTS	26.50	7.30	72	3	LNCaP	Human prostate cancer	Prostate	Xanthohumol
Deeb et al. [62]	MTS	29.00	7.30	72	3	DU145	Human prostate cancer	Prostate	Xanthohumol
Deeb et al. [62]	MTS	31.00	7.30	72	3	C4-2	Human prostate cancer	Prostate	Xanthohumol
Deeb et al. [62]	MTS	25.10	7.30	72	3	PC-3	Human prostate cancer	Prostate	Xanthohumol
Negrão et al. [63]	MTT	24.00	6.00	24	3	HUVEC	Human umbilical vein endothelial	Non-cancer	Xanthohumol
Negrão et al. [63]	MTT	12.00	6.00	24	3	HASMC	Human aortic smooth muscle	Non-cancer	Xanthohumol
Negrão et al. [63]	MTT	28.00	4.05	24	3	HUVEC	Human umbilical vein endothelial	Non-cancer	Isoxanthohumol
Wesołowska et al. [64]	SRB	33	3.42	72	3	LoVo	Human colon cancer	Colon	8-prenylnaringenin
Wesołowska et al. [64]	SRB	55	3.42	72	3	LoVo	Human colon cancer	Colon	8-prenylnaringenin
Strathmann et al. [65]	SRB	6.7	0.2	72	3	BPH-1	Human benign prostatic hyperplasia	Non-cancer	Xanthohumol
Dorn et al. [66]	XTT	20.00	5.00	72	3	HepG2	Human liver cancer	Liver	Xanthohumol
Dorn et al. [66]	XTT	15.00	5.00	72	3	Huh7	Human liver cancer	Liver	Xanthohumol
Mendes et al. [67]	SRB	26	27.31	24	3	3T3-L1	Murine preadipocyte	Non-cancer	Xanthohumol
Mendes et al. [67]	SRB	12	27.31	48	3	3T3-L1	Murine preadipocyte	Non-cancer	Xanthohumol
Mendes et al. [67]	SRB	17	27.31	72	3	3T3-L1	Murine preadipocyte	Non-cancer	Xanthohumol
Ho et al. [68]	MTT	166.00	3.00	24	3	HA22T/VGH	Human liver cancer	Liver	Xanthohumol
Ho et al. [68]	MTT	108.00	5.00	24	3	Hep3B	Human liver cancer	Liver	Xanthohumol
Ho et al. [68]	MTT	211.00	6.00	24	3	AML12	Murine normal liver	Non-cancer	Xanthohumol
Monteghirfo et al. [69]	MTT	10.00	1.50	48	3	K562	Human leukemia	Leukemia	Xanthohumol
Monteghirfo et al. [69]	MTT	16.00	1.50	48	3		Mononuclear cells from CML patients	Leukemia	Xanthohumol
Monteghirfo et al. [69]	MTT	5.40	1.50	72	3	K562	Human leukemia	Leukemia	Xanthohumol
Koo et al. [70]	MTT	8.60	1.15	24	3	B16-F10	Murine melanoma	Melanoma	Xanthohumol
Lee et al. [71]	SRB	12.13	3.43	48	3	A549	Human lung cancer	Lung	Xanthohumol
Lee et al. [71]	SRB	10.15	8.92	48	3	HCT15	Human colon cancer	Colon	Xanthohumol
Lee et al. [71]	SRB	14.39	8.92	48	3	SK-MEL-2	Human melanoma	Melanoma	Xanthohumol
Lee et al. [71]	SRB	16	1.03	48	3	SK-OV-3	Human ovarian cancer	Ovarian	Xanthohumol
Lee et al. [71]	SRB	100.17	3.42	48	3	HCT15	Human colon cancer	Colon	8-prenylnaringenin
Lee et al. [71]	SRB	66.39	11.31	48	3	A549	Human lung cancer	Lung	8-prenylnaringenin
Lee et al. [71]	SRB	102.23	11.31	48	3	SK-MEL-2	Human melanoma	Melanoma	8-prenylnaringenin
Lee et al. [71]	SRB	75.2	10.14	48	3	SK-OV-3	Human ovarian cancer	Ovarian	8-prenylnaringenin
Lee et al. [71]	SRB	63.48	4	48	3	HCT15	Human colon cancer	Colon	Isoxanthohumol
Lee et al. [71]	SRB	77.59	4.2	48	3	A549	Human lung cancer	Lung	Isoxanthohumol
Lee et al. [71]	SRB	40.34	19.59	48	3	SK-MEL-2	Human melanoma	Melanoma	Isoxanthohumol
Lee et al. [71]	SRB	27.93	1.65	48	3	SK-OV-3	Human ovarian cancer	Ovarian	Isoxanthohumol
Yang et al. [72]	MTS	75.00	14.14	24	8	3T3-L1	Murine preadipocyte	Non-cancer	Xanthohumol
Yang et al. [72]	MTS	53.00	9.90	48	8	3T3-L1	Murine preadipocyte	Non-cancer	Xanthohumol
Monteiro et al. [73]	SRB	7.1	4.6	72	9	Sk-Br-3	Human breast cancer	Breast	Xanthohumol
Monteiro et al. [73]	SRB	22.6	9.7	72	9	Sk-Br-3	Human breast cancer	Breast	8-prenylnaringenin
Monteiro et al. [73]	SRB	41	12.68	72	9	Sk-Br-3	Human breast cancer	Breast	Isoxanthohumol
Plazar et al. [74]	MTT	75.00	3.48	24	5	HepG2	Human liver cancer	Liver	Xanthohumol
Delmulle et al. [75]	WST-1	13.20	1.10	48	3	PC-3	Human prostate cancer	Prostate	Xanthohumol
Delmulle et al. [75]	WST-1	12.30	1.10	48	3	DU145	Human prostate cancer	Prostate	Xanthohumol
Delmulle et al. [75]	WST-1	18.40	1.20	48	3	PC-3	Human prostate cancer	Prostate	6-prenylnaringenin
Delmulle et al. [75]	WST-1	29.10	1.10	48	3	DU145	Human prostate cancer	Prostate	6-prenylnaringenin
Delmulle et al. [75]	WST-1	33.50	1.00	48	3	PC-3	Human prostate cancer	Prostate	8-prenylnaringenin
Delmulle et al. [75]	WST-1	43.10	1.20	48	3	DU145	Human prostate cancer	Prostate	8-prenylnaringenin
Delmulle et al. [75]	WST-1	45.20	1.10	48	3	PC-3	Human prostate cancer	Prostate	Isoxanthohumol
Delmulle et al. [75]	WST-1	47.40	1.10	48	3	DU145	Human prostate cancer	Prostate	Isoxanthohumol
Delmulle et al. [75]	WST-1	49.90	1.00	48	3	PC-3	Human prostate cancer	Prostate	Desmethylxanthohumol
Delmulle et al. [75]	WST-1	53.80	1.10	48	3	DU145	Human prostate cancer	Prostate	Desmethylxanthohumol
Colgate et al. [76]	MTT	24.00	7.30	48	4	PC-3	Human prostate cancer	Prostate	Xanthohumol
Colgate et al. [76]	MTT	5.00	6.00	48	4	BPH-1	Human benign prostatic hyperplasia	Non-cancer	Xanthohumol
Dietz et al. [77]	Crystal Violet	30.70	7.6	48	3	Hepa 1c1c7	Murine liver cancer	Liver	Xanthohumol
Dietz et al. [77]	Crystal Violet	30.70	2.9	48	3	Hepa 1c1c7	Murine liver cancer	Liver	Isoxanthohumol
Pan et al. [78]	SRB	4.1	0.9	24	3	HCT116	Human colon cancer	Colon	Xanthohumol
Pan et al. [78]	SRB	3.6	0.6	48	3	HCT116	Human colon cancer	Colon	Xanthohumol
Pan et al. [78]	SRB	2.6	0.1	72	3	HCT116	Human colon cancer	Colon	Xanthohumol
Gerhauser et al. [3]	Crystal Violet	7.40	1.4	48	3	Hepa 1c1c7	Murine liver cancer	Liver	Xanthohumol
Gerhauser et al. [3]	Crystal Violet	29.90	1.9	48	3	Hepa 1c1c7	Murine liver cancer	Liver	Isoxanthohumol
Miranda et al. [79]	SRB	13.3	1.16	48	4	MCF-7	Human breast cancer	Breast	Xanthohumol
Miranda et al. [79]	SRB	46	8.92	48	4	HT-29	Human colon cancer	Colon	Xanthohumol
Miranda et al. [79]	SRB	0.52	1.03	48	4	A2780	Human ovarian cancer	Ovarian	Xanthohumol
Miranda et al. [79]	SRB	3.47	1.16	96	4	MCF-7	Human breast cancer	Breast	Xanthohumol
Miranda et al. [79]	SRB	51.1	8.92	96	4	HT-29	Human colon cancer	Colon	Xanthohumol
Miranda et al. [79]	SRB	5.22	1.03	96	4	A2780	Human ovarian cancer	Ovarian	Xanthohumol
Miranda et al. [79]	SRB	15.3	12.68	48	4	MCF-7	Human breast cancer	Breast	Isoxanthohumol
Miranda et al. [79]	SRB	62.5	4.15	48	4	HT-29	Human colon cancer	Colon	Isoxanthohumol
Miranda et al. [79]	SRB	18	1.65	48	4	A2780	Human ovarian cancer	Ovarian	Isoxanthohumol
Miranda et al. [79]	SRB	4.69	12.68	96	4	MCF-7	Human breast cancer	Breast	Isoxanthohumol
Miranda et al. [79]	SRB	25.7	1.65	96	4	A2780	Human ovarian cancer	Ovarian	Isoxanthohumol
Miranda et al. [79]	SRB	57.8	4.15	96	4	HT-29	Human colon cancer	Colon	Isoxanthohumol

**Table 2:** Meta-regression analysis of IC50 values of XN derived from tetrazolium salt, SRB and crystal violet (CV) assays, on cancer and non-cancer cells, for different incubation times (24, 48 and 72 h).

Time (h)	Cancer		Non-Cancer	
	*p*-Value	Number of Studies	*p*-Value	Number of Studies
24	0.777	37	0.517	9
48	0.350	46	0.356	12
72	0.702	52	0.913	9

**Table 3:** Standardized mean difference-based meta-analysis of IC_50_ values of individual chemical compounds obtained with tetrazolium salt-based and crystal violet (CV) assays over 48 h incubation time on specific cancer cell lines.

Compound	Time (h)	Number of Experiments	Number of Studies	SMD	95% CI	*p*-Value	Type of Cancer
Xanthohumol	48	12	2	0.62	0.00, 1.79	0.30	Melanoma
Isoxanthohumol	48	18	3	1.00	0.18, 4.98	0.16	Melanoma
8-prenylnaringenin	48	12	2	0.88	0.00, 3.55	0.52	Melanoma

**Table 4:** Random-effects meta-analysis of IC_50_ values (obtained from all assays, collectively) along with 95% confidence interval, *p*-value and I-squared (I^2^) for all groups of compounds on different types of cells (cancer/non-cancer) and for different incubation times.

Group of Compounds	Time	Number of Studies	Type of Cancer	IC_50_ (μΜ/ μg/mL) *	95% CI	*p*-Value	I^2^ (%)
**Chalcones**	24	5	Glioblastoma	60.44	55.13, 65.75	0.000	51.6
	24	2	Neck	50.80	40.61, 60.99	0.000	67.2
	24	4	Gastric	42.75	0.00, 88.89	0.069	99.2
	24	6	Liver	88.08	52.80, 123.35	0.000	99.7
	24	2	Pancreas	18.85	1.50, 36.20	0.033	88.7
	24	5	Colon	33.83	11.56, 56.11	0.003	99.9
	24	7	Breast	48.97	29.38, 68.57	0.000	98.1
	24	37	**Cancer**	**52.16**	**42.66, 61.66**	**0.000**	**99.8**
	24	9	**Non-cancer**	**90.03**	**35.94, 144.11**	**0.001**	**99.9**
**Chalcones**	48	3	Leukemia	15.22	9.55, 20.89	0.000	98.0
	48	2	Myeloma	35.99	8.28, 63.69	0.011	95.6
	48	5	Melanoma	12.54	8.89, 16.20	0.000	94.3
	48	4	Neck	18.93	14.11, 23.74	0.000	26.4
	48	3	Lung	15.50	3.18, 27.81	0.014	98.5
	48	4	Liver	27.69	7.89, 47.48	0.006	99.7
	48	2	Pancreas	8.60	2.76, 14.44	0.004	0.0
	48	6	Colon	17.02	8.62, 25.41	0.000	96.7
	48	2	Bone	72.79	0.00, 157.86	0.093	99.9
	48	7	Breast	21.75	13.26, 30.24	0.000	97.1
	48	2	Cervix	21.27	0.00, 46.85	0.103	97.2
	48	2	Ovarian	8.26	0.00, 23.43	0.286	99.7
	48	5	Prostate	30.67	10.98, 50.37	0.002	99.9
	48	50	**Cancer**	**22.54**	**18.06, 27.01**	**0.000**	**99.7**
	48	14	**Non-cancer**	**65.53**	**48.75, 82.32**	**0.000**	**99.9**
**Chalcones**	72	3	Leukemia	6.59	3.26, 9.91	0.000	92.5
	72	2	Neck	18.65	12.81, 24.49	0.000	0.0
	72	2	Lung	17.02	9.82, 24.21	0.000	93.2
	72	4	Liver	39.79	0.00, 83.02	0.071	99.7
	72	5	Pancreas	11.86	7.16, 16.56	0.000	59.8
	72	10	Colon	23.29	19.58, 27.00	0.000	99.8
	72	17	Breast	10.97	9.79, 12.14	0.000	94.9
	72	4	Ovarian	5.73	1.90, 9.57	0.003	98.7
	72	12	Prostate	13.51	11.37, 15.64	0.000	99.8
	72	63	**Cancer**	**15.93**	**14.42, 17.43**	**0.000**	**99.8**
	72	11	**Non-cancer**	**33.89**	**15.15, 52.62**	**0.000**	**99.9**
**Flavones**	24	3	Colon	97.16	31.77, 162.55	0.004	99.6
	24	2	Breast	109.68	99.90, 119.45	0.000	61.7
	24	5	**Cancer**	**102.26**	**62.78, 141.74**	**0.000**	**99.3**
**Flavones**	48	12	Melanoma	36.81	29.99, 43.62	0.000	98.9
	48	3	Lung	65.83	47.94, 83.71	0.000	96.1
	48	2	Liver	30.14	28.34, 31.94	0.000	0.0
	48	4	Colon	74.05	56.91, 91.19	0.000	98.7
	48	3	Ovarian	37.41	25.11, 49.72	0.000	98.6
	48	6	Prostate	36.12	27.47, 44.77	0.000	99.6
	48	32	**Cancer**	**43.76**	**38.89, 48.64**	**0.000**	**99.4**
**Flavones**	72	9	Colon	50.55	33.01, 68.09	0.000	99.7
	72	2	Uterus	23.16	9.04, 37.27	0.001	95.2
	72	13	Breast	35.55	27.15, 43.96	0.000	97.9
	72	6	Ovarian	36.19	26.65, 45.74	0.000	98.3
	72	7	Prostate	62.84	55.13, 70.54	0.000	80.5
	72	39	**Cancer**	**42.95**	**36.91, 48.99**	**0.000**	**99.4**
	72	7	**Non-cancer**	**46.96**	**31.08, 62.84**	**0.000**	**96.9**
**α-acids**	48	2	**Non-cancer**	**30.67**	**28.10, 33.24**	**0.000**	**75.4**
**α-acids**	72	7	Breast	16.06	10.24, 21.88	0.000	63.7
	72	7	**Cancer**	**16.06**	**10.24, 21.88**	**0.000**	**63.7**
**β-acids**	48	2	Prostate	9.75	8.28, 11.22	0.000	92.6
	48	4	**Cancer**	**8.20**	**4.77, 11.63**	**0.000**	**99.4**
	48	2	**Non-cancer**	**3.10**	**2.12, 4.08**	**0.000**	**99.3**
**β-acids**	72	2	Prostate	5.00	4.60, 5.40	0.000	0.0
	72	2	**Cancer**	**5.00**	**4.60, 5.40**	**0.000**	**0.0**
**Hops crude extract** **	24	3	**Non-cancer**	**57.90**	**41.20, 74.60**	**0.000**	**99.9**
**Hops crude extract** **	72	2	Liver	16.57	0.00, 35.65	0.089	99.2
	72	5	**Cancer**	**35.23**	**15.20, 55.26**	**0.001**	**99.5**
	72	3	**Non-cancer**	**43.80**	**0.00, 87.78**	**0.051**	**99.9**

* IC_50_ is measured in μM for chemical compounds and μg/mL for crude extracts. “Cancer” denotes meta-analysis results for collectively all cancer cell lines. ** Full extraction details are reported in Table 1; no two studies employed identical extraction conditions. Bold letters denote head categories of compounds or types of cell lines.

**Table 5:** Meta-regression analysis of IC_50_ values (obtained from all assays, collectively) of various compounds on cancer and non-cancer cells for different incubation times. “Cancer” denotes meta-analysis results for all cancer cell lines collectively.

	24 h		48 h		72 h	
Compounds	*p*-Value	Number of Studies (Cancer/Non-Cancer)	*p*-Value	Number of Studies (Cancer/Non-Cancer)	*p*-Value	Number of Studies (Cancer/Non-Cancer)
**Chalcones**	**0.049**	**(37/9)**	**0.000**	**(50/14)**	**0.019**	**(63/11)**
Xanthohumol	0.049	(37/9)	0.000	(46/12)	0.088	(52/9)
α,β-dihydroxanthohumol					0.046	(11/2)
Desmethylxanthohumol			0.566	(4/2)		
**Flavones**					**0.712**	**(39/7)**
8-prenylnaringenin					0.777	(12/2)
6-prenylnaringenin					0.947	(8/2)
Isoxanthohumol					0.760	(19/3)
**β-acids**			**0.129**	**(4/2)**		
Lupulone			0.129	(4/2)		

Bold denote head categories of compounds along with their results.

## Data Availability

All data used or produced in this research are within the tables, figures or Supplementary Materials of this manuscript.

## Supplementary Materials

The following supporting information can be downloaded at: https://www.mdpi.com/article/10.3390/ph18081139/s1, Figure S1: Meta-regression analysis of IC_50_ values; Figure S2: Forest plots of meta-analysis of IC_50_ values of Xanthohumol; Figure S3: Forest plots of meta-analysis of IC_50_ values of Chalcones and Flavones; Table S1: Number of studies of present meta-analysis; Table S2: Random-effects model meta-analysis for the different assays and different types of cancer; Table S3: Random effects meta-analysis of IC_50_ values of ΧΝ; Table S4: Characteristics of studies that were used in meta-analysis of SMDs; Table S5: Random effects meta-analysis of IC_50_ values of ΧΝ on different types of cancer and non-cancer cells; Table S6: Meta-regression analysis of IC_50_ values of all chemical compounds on cancer and non-cancer cells for all incubation time points.

## Abbreviations

The following abbreviations are used in this manuscript:

MTT	3-[4,5-dimethylthiazol-2-yl]-2,5 diphenyl tetrazolium bromide
SRB	Sulfohodamine B
XTT	2,3-bis-(2-methoxy-4-nitro-5-sulfophenyl)-2H-tetrazolium-5-carboxanilide
MTS	3-(4,5-dimethylthiazol-2-yl)-5-(3-carboxymethoxyphenyl)-2-(4-sulfophenyl)-2H-tetrazolium
WST	Water-Soluble Tetrazolium Salt
CV	Crystal Violet
PRISMA	Preferred Reporting Items for Systematic Reviews and Meta-Analyses
SMD	Standardized Mean Difference
IC_50_	Half Maximal Inhibitory Concentration
XN	Xanthohumol
IXN	Isoxanthohumol
DMX	Desmethylxanthohumol
8-PN	8-prenylnaringenin
6-PN	6-prenylnaringenin
