# Supplementary material for: Humulus lupulus (Hop)-Derived Chemical Compounds Present Antiproliferative Activity on Various Cancer Cell Types: A Meta-Regression Based Panoramic Meta-Analysis
Source: Pharmaceuticals (Basel). 2025 Jul 31;18(8):1139. doi: 10.3390/ph18081139 (PMC12388921; doi:10.3390/ph18081139)
Supplement: Supplementary file 1 [file pharmaceuticals-18-01139-s001.zip › sup figure legends.pdf]

Supplementary Figure S1

Meta-regression analysis of IC_{50} values measured by SRB, Tetrazolium dye, and CV assays for cancer (A, B, C) and non-cancer (D, E, F) cell lines for 24h (A , D), 48 h (B, E) and 72h (C, F) of incubation.

Supplementary Figure S2

Forest plots of meta-analysis of IC_{50} values of Xanthohumol stratified according to cell types (cancer and non-cancer) for 24h (A), 48h (B) and 72h (C) of incubation

Supplementary Figure S3

Forest plots of meta-analysis of IC_{50} values of Chalcones (A, B, C) and flavones (D, E F) for cancer cells and for 24h (A, D), 48h (B, E) and 72h (C, F) of incubation
