# Supplementary material for: Humulus lupulus (Hop)-Derived Chemical Compounds Present Antiproliferative Activity on Various Cancer Cell Types: A Meta-Regression Based Panoramic Meta-Analysis
Source: Pharmaceuticals (Basel). 2025 Jul 31;18(8):1139. doi: 10.3390/ph18081139 (PMC12388921; doi:10.3390/ph18081139)
Supplement: Supplementary file 1 [file pharmaceuticals-18-01139-s001.zip › SUP_TABLE 1.pdf]

Supplementary Table S1. Number of studies of present meta-analysis according to different types of cancer and compounds of *Humulus lupulus*.

Type of cancer	Number of studies	Compounds	Number of studies
Leukemia	7	Xanthohumol	172
Myeloma	4	Isoxanthohumol	46
Melanoma	18	8-prenylnaringenin	28
Glioblastoma	8	α,β-dihydroxanthohumol	13
Neck	8	6-prenylnaringenin	13
Thyroid	3	Hop/crude extract	12
Esophagus	3	Naringenin	10
Lung	11	Lupulone	8
Gastric	4	Desmethylxanthohumol	6
Liver	19	Tetrahydro Iso-Alpha Acids	2
Pancreas	9	α-acids	2
Colon	40	Humulone	2
Uterus	2	Iso-α-acids	2
Bone	4	Dihydro-iso-alpha acids	1
Breast	62		
Cervix	5		
Ovarian	19		
Prostate	36		
