# Supplementary material for: Humulus lupulus (Hop)-Derived Chemical Compounds Present Antiproliferative Activity on Various Cancer Cell Types: A Meta-Regression Based Panoramic Meta-Analysis
Source: Pharmaceuticals (Basel). 2025 Jul 31;18(8):1139. doi: 10.3390/ph18081139 (PMC12388921; doi:10.3390/ph18081139)
Supplement: Supplementary file 1 [file pharmaceuticals-18-01139-s001.zip › SUP_TABLE 2.pdf]

Supplementary Table S2. Random-effects model meta-analysis for the different assays and different types of cancer. Listed information includes the chemical compound, the incubation time, the overall effect size (IC$_{50}$) along with 95% confidence interval, p-value and I-squared (I^2).

Assay	Compound	Time	Number of studies	Type of cancer	IC$_{50}$(µM/µg/ml)*	95% CI	p-value	I^2 (%)
Tetrazolium salt	Xanthohumol	24	5	Glioblastoma	60.44	55.13, 65.75	0.000	51.6
Tetrazolium salt	Xanthohumol	24	2	Neck	50.80	40.61, 60.99	0.000	67.2
Tetrazolium salt	Xanthohumol	24	4	Gastric	42.75	0.00, 88.89	0.069	99.2
Tetrazolium salt	Xanthohumol	24	4	Liver	116.53	73.42, 159.64	0.000	99.8
Tetrazolium salt	Xanthohumol	24	2	Pancreas	18.85	1.50, 36.20	0.033	88.7
Tetrazolium salt	Xanthohumol	24	7	Breast	48.97	29.38, 68.57	0.000	98.1
Tetrazolium salt	Xanthohumol	24	28	**Cancer**	**54.08**	**36.49, 71.66**	**0.000**	**99.8**
Tetrazolium salt	Xanthohumol	24	8	**Non-cancer**	**97.76**	**40.40, 155.12**	**0.001**	**99.9**
Tetrazolium salt	Xanthohumol	48	3	Leukemia	15.22	9.55, 20.89	0.000	98.0
Tetrazolium salt	Xanthohumol	48	2	Myeloma	35.99	8.28, 63.69	0.011	95.6
Tetrazolium salt	Xanthohumol	48	2	Melanoma	11.84	5.67, 18.02	0.000	98.1
Tetrazolium salt	Xanthohumol	48	4	Neck	18.93	14.11, 23.74	0.000	26.4
Tetrazolium salt	Xanthohumol	48	2	Pancreas	8.60	2.76, 14.44	0.004	0.0
Tetrazolium salt	Xanthohumol	48	6	Breast	23.42	16.21, 30.63	0.000	91.3
Tetrazolium salt	Xanthohumol	48	3	Prostate	13.86	11.36, 16.37	0.000	80.7
Tetrazolium salt	Xanthohumol	48	28	**Cancer**	**17.64**	**15.00, 20.28**	**0.000**	**97.2**
Tetrazolium salt	Xanthohumol	48	10	**Non-cancer**	**53.89**	**42.06, 65.73**	**0.000**	**99.8**
Tetrazolium salt	Xanthohumol	72	3	Leukemia	6.59	3.26, 9.91	0.000	92.5
Tetrazolium salt	Xanthohumol	72	2	Neck	18.65	12.81, 24.49	0.000	0.0
Tetrazolium salt	Xanthohumol	72	3	Liver	45.89	0.00, 107.03	0.141	99.8
Tetrazolium salt	Xanthohumol	72	5	Pancreas	11.863	7.16, 16.56	0.000	59.8
Tetrazolium salt	Xanthohumol	72	3	Breast	18.768	10.26, 7.28	0.000	99.8
Tetrazolium salt	Xanthohumol	72	4	Prostate	27.900	23.77, 2.03	0.000	0.0
Tetrazolium salt	Xanthohumol	72	23	**Cancer**	**19.848**	**13.42, 26.28**	**0.000**	**99.1**
Tetrazolium salt	Xanthohumol	72	2	**Non-cancer**	**34.53**	**6.44, 62.63**	**0.016**	**98.5**
Tetrazolium salt	Xanthohumol	96	2	Myeloma	23.32	0.00, 52.88	0.122	96.1
Tetrazolium salt	Xanthohumol	96	3	**Cancer**	**18.48**	**0.00, 38.00**	**0.064**	**94.0**
Tetrazolium salt	Hop/crude extract	24	3	**Non-cancer**	**57.90**	**41.20, 74.60**	**0.000**	**99.9**
Tetrazolium salt	Hop/crude extract	72	2	Liver	16.57	0.00, 35.65	0.089	99.2
Tetrazolium salt	Hop/crude extract	72	5	**Cancer**	**35.23**	**15.20, 55.26**	**0.001**	**99.5**
Tetrazolium salt	Hop/crude extract	72	3	**Non-cancer**	**43.80**	**0.00, 87.78**	**0.051**	**99.9**
Tetrazolium salt	6-prenylnaringenin	48	2	Prostate	23.75	13.27, 34.24	0.000	99.2
Tetrazolium salt	6-prenylnaringenin	48	2	**Cancer**	**23.75**	**13.27, 34.24**	**0.000**	**99.2**
Tetrazolium salt	8-prenylnaringenin	24	2	Colon	118.34	23.60, 213.09	0.014	99.5
Tetrazolium salt	8-prenylnaringenin	24	3	**Cancer**	**117.23**	**62.48, 171.97**	**0.000**	**99.0**
Tetrazolium salt	8-prenylnaringenin	48	2	Melanoma	34.50	21.72, 47.29	0.000	97.0

Tetrazolium salt	8-prenylnaringenin	48	2	Prostate	38.292	28.89, 47.70	0.000	99.1
Tetrazolium salt	8-prenylnaringenin	48	5	**Cancer**	**43.051**	**35.57, 50.53**	**0.000**	**99.2**
Tetrazolium salt	Isoxanthohumol	48	3	Melanoma	24.711	20.57, 28.85	0.000	77.9
Tetrazolium salt	Isoxanthohumol	48	2	Prostate	46.300	44.14, 48.46	0.000	83.3
Tetrazolium salt	Isoxanthohumol	48	7	Cancer	38.222	27.50, 48.94	0.000	99.6
Tetrazolium salt	Lupulone	48	2	Prostate	9.750	8.28, 11.22	0.000	92.6
Tetrazolium salt	Lupulone	48	4	**Cancer**	**8.201**	**4.77, 11.63**	**0.000**	**99.4**
Tetrazolium salt	Lupulone	48	2	**Non-cancer**	**3.10**	**2.12, 4.08**	**0.000**	**99.3**
Tetrazolium salt	Lupulone	72	2	Prostate	5.00	4.60, 5.40	0.000	0.0
Tetrazolium salt	Lupulone	72	2	**Cancer**	**5.00**	**4.60, 5.40**	**0.000**	**0.0**
Tetrazolium salt	Humulone	48	2	**Non-cancer**	**30.67**	**28.10, 33.24**	**0.000**	**75.4**
Tetrazolium salt	Desmethylxanthohumol	48	2	Prostate	51.84	48.02, 55.66	0.000	95.2
Tetrazolium salt	Desmethylxanthohumol	48	4	**Cancer**	**71.35**	**53.77, 88.93**	**0.000**	**99.7**
Tetrazolium salt	Desmethylxanthohumol	48	2	**Non-cancer**	**103.49**	**0.00, 250.49**	**0.168**	**100**
SRB	Xanthohumol	24	2	Liver	31.29	19.73, 42.86	0.000	99.5
SRB	Xanthohumol	24	3	Colon	31.70	2.69, 60.70	0.032	99.9
SRB	Xanthohumol	24	8	**Cancer**	**46.80**	**33.11, 60.49**	**0.000**	99.9
SRB	Xanthohumol	48	2	Lung	18.95	5.87, 32.03	0.005	97.7
SRB	Xanthohumol	48	3	Colon	19.75	0.00, 45.72	0.136	97.8
SRB	Xanthohumol	48	2	Ovarian	8.26	0.00, 23.43	0.286	99.7
SRB	Xanthohumol	48	11	**Cancer**	**20.80**	**13.27, 28.32**	**0.000**	**99.7**
SRB	Xanthohumol	48	2	**Non-cancer**	**81.42**	**0.00, 215.86**	**0.235**	**98.5**
SRB	Xanthohumol	72	5	Colon	31.20	26.04, 36.36	0.000	99.9
SRB	Xanthohumol	72	10	Breast	9.71	8.64, 10.77	0.000	89.2
SRB	Xanthohumol	72	2	Ovarian	5.14	0.00, 11.17	0.095	99.1
SRB	Xanthohumol	72	5	Prostate	8.80	7.24, 10.36	0.000	88.0
SRB	Xanthohumol	72	26	**Cancer**	**14.60**	**12.57, 16.63**	**0.000**	**99.6**
SRB	Xanthohumol	72	7	**Non-cancer**	**31.03**	**2.45, 59.62**	**0.033**	**99.8**
SRB	Xanthohumol	96	3	**Cancer**	**15.53**	**8.57, 22.49**	**0.000**	**98.2**
SRB	6-prenylnaringenin	72	3	Breast	39.41	16.32, 62.51	0.001	98.4
SRB	6-prenylnaringenin	72	2	Ovarian	62.69	25.88, 99.50	0.001	92.5
SRB	6-prenylnaringenin	72	2	Prostate	79.23	71.76, 86.70	0.000	0.0
SRB	6-prenylnaringenin	72	8	**Cancer**	**57.66**	**38.86, 76.47**	**0.000**	**98.0**
SRB	6-prenylnaringenin	72	2	**Non-cancer**	**60.78**	**0.00,155.19**	**0.207**	**97.6**
SRB	8-prenylnaringenin	48	4	**Cancer**	**86.40**	**69.27, 103.53**	**0.000**	**92.2**
SRB	8-prenylnaringenin	72	3	Colon	59.30	25.15, 93.45	0.001	99.6
SRB	8-prenylnaringenin	72	4	Breast	40.73	21.17, 60.29	0.000	97.0
SRB	8-prenylnaringenin	72	2	Ovarian	46.06	6.41, 85.71	0.023	97.9
SRB	8-prenylnaringenin	72	2	Prostate	56.88	48.02, 65.74	0.000	59.5
SRB	8-prenylnaringenin	72	11	**Cancer**	**49.57**	**34.42, 64.72**	**0.000**	**98.8**
SRB	8-prenylnaringenin	72	2	**Non-cancer**	**56.91**	**0.00, 122.37**	**0.088**	**97.7**

SRB	Isoxanthohumol	48	2	Colon	62.94	59.91, 65.96	0.000	0.0
SRB	Isoxanthohumol	48	2	Ovarian	22.95	13.22, 32.69	0.000	98.4
SRB	Isoxanthohumol	48	7	**Cancer**	**43.90**	**26.91, 60.89**	**0.000**	**99.4**
SRB	Isoxanthohumol	72	4	Colon	38.73	16.87, 60.59	0.001	99.8
SRB	Isoxanthohumol	72	2	Uterus	23.16	9.04, 37.27	0.001	95.2
SRB	Isoxanthohumol	72	6	Breast	30.38	21.08, 39.68	0.000	95.5
SRB	Isoxanthohumol	72	2	Ovarian	9.81	6.16, 13.45	0.000	93.1
SRB	Isoxanthohumol	72	3	Prostate	58.52	52.08, 64.96	0.000	33.7
SRB	Isoxanthohumol	72	18	**Cancer**	**33.50**	**26.96, 40.04**	**0.000**	**99.4**
SRB	Isoxanthohumol	72	3	**Non-cancer**	**38.50**	**15.59, 61.41**	**0.000**	**97.4**
SRB	Isoxanthohumol	96	3	**Cancer**	**29.95**	**4.38, 55.52**	**0.022**	**99.1**
SRB	Naringenin	72	3	Breast	129.24	113.62, 144.87	0.000	16.2
SRB	Naringenin	72	2	Ovarian	101.75	94.76, 108.73	0.000	26.2
SRB	Naringenin	72	2	Prostate	150.69	114.03, 187.34	0.000	85.9
SRB	Naringenin	72	8	**Cancer**	**127.15**	**110.50, 143.80**	**0.000**	**94.0**
SRB	Naringenin	72	2	**Non-cancer**	**146.16**	**78.72, 213.60**	**0.000**	**74.2**
SRB	α,β-dihydroxanthohumol	72	2	Colon	42.23	0.00, 103.13	0.174	96.4
SRB	α,β-dihydroxanthohumol	72	4	Breast	9.17	8.59, 9.76	0.000	1.4
SRB	α,β-dihydroxanthohumol	72	2	Ovarian	6.59	0.00, 16.16	0.179	97.6
SRB	α,β-dihydroxanthohumol	72	3	Prostate	14.43	12.14, 16.72	0.000	0.0
SRB	α,β-dihydroxanthohumol	72	11	**Cancer**	**11.58**	**8.22, 14.93**	**0.000**	**97.9**
SRB	α,β-dihydroxanthohumol	72	2	**Non-cancer**	**43.02**	**0.00, 99.74**	**0.137**	**99.5**
Crystal Violet	Xanthohumol	48	2	Melanoma	12.94	7.26, 18.62	0.000	91.7
Crystal Violet	Xanthohumol	48	2	Liver	18.65	0.00, 41.47	0.109	96.3
Crystal Violet	Xanthohumol	48	3	Colon	14.25	9.57, 18.92	0.000	68.7
Crystal Violet	Xanthohumol	48	7	**Cancer**	**14.39**	**10.44, 18.33**	**0.000**	**91.7**
Crystal Violet	Xanthohumol	72	3	Colon	12.06	6.31, 17.81	0.000	95.0
Crystal Violet	Xanthohumol	72	3	**Cancer**	**12.06**	**6.31, 17.81**	**0.000**	**95.0**
Crystal Violet	8-prenylnaringenin	48	2	Melanoma	42.46	33.10, 51.83	0.000	25.4
Crystal Violet	Isoxanthohumol	48	3	Melanoma	29.18	18.68, 39.70	0.000	94.2
Crystal Violet	Isoxanthohumol	48	2	Liver	30.14	28.34, 31.94	0.000	0.0
Crystal Violet	Isoxanthohumol	48	5	**Cancer**	**29.48**	**24.69, 34.27**	**0.000**	**92.2**

*IC_{50} is measured in μM for the chemical compounds and μg/ml for the crude extracts.

"Cancer" denotes meta-analysis results for collectively all cancer cell lines
