# Supplementary material for: Humulus lupulus (Hop)-Derived Chemical Compounds Present Antiproliferative Activity on Various Cancer Cell Types: A Meta-Regression Based Panoramic Meta-Analysis
Source: Pharmaceuticals (Basel). 2025 Jul 31;18(8):1139. doi: 10.3390/ph18081139 (PMC12388921; doi:10.3390/ph18081139)
Supplement: Supplementary file 1 [file pharmaceuticals-18-01139-s001.zip › SUP_TABLE 3.pdf]

Supplementary Table S3. Random effects meta-analysis of IC_{50} values (obtained from Tetrazolium salt, SRB and CV assays) of Xanthohumol on different types of cells for different incubation time points.

Assay	Time	Number of studies	Type of cancer	IC_{50} (µM)	95% CI	p-value
Tetrazolium salt	24	28	Cancer	54.08	36.49, 71.66	0.000
Tetrazolium salt	24	8	Non-cancer	97.76	40.40, 155.12	0.001
Tetrazolium salt	48	28	Cancer	17.64	15.00, 20.28	0.000
Tetrazolium salt	48	10	Non-cancer	53.89	42.06, 65.73	0.000
Tetrazolium salt	72	23	Cancer	19.85	13.42, 26.28	0.000
Tetrazolium salt	72	2	Non-cancer	34.53	6.44, 62.63	0.016
SRB	24	8	Cancer	46.80	33.11, 60.49	0.000
SRB	48	2	Non-cancer	81.41	0.00, 215.86	0.235
SRB	48	11	Cancer	20.78	13.27, 28.32	0.000
SRB	72	26	Cancer	14.60	12.57, 16.63	0.000
SRB	72	7	Non-cancer	31.03	2.45, 59.62	0.033
Crystal Violet	48	7	Cancer	14.38	10.44, 18.33	0.000
Crystal Violet	72	3	Cancer	12.06	6.31, 17.81	0.000

"Cancer" denotes meta-analysis results for collectively all cancer cell lines
