# Supplementary material for: Humulus lupulus (Hop)-Derived Chemical Compounds Present Antiproliferative Activity on Various Cancer Cell Types: A Meta-Regression Based Panoramic Meta-Analysis
Source: Pharmaceuticals (Basel). 2025 Jul 31;18(8):1139. doi: 10.3390/ph18081139 (PMC12388921; doi:10.3390/ph18081139)
Supplement: Supplementary file 1 [file pharmaceuticals-18-01139-s001.zip › SUP_TABLE 4.pdf]

Supplementary Table S4. Characteristics of studies that were used in meta-analysis of standardized mean differences (SMDs) of IC_{50} values obtained with MTT and CV assays.

Study	Time (hours)	Number of experiments (MTT)	IC_{50}(µM) (MTT)	SD (MTT)	Number of experiments (CV)	IC_{50}(µM) CV	SD (CV)	Type of cancer	Cell line	Compound
Krajnović et al. (2016)	48	3	8.70	0.99	3	9.97	2.32	Murine melanoma	B16	Xanthohumol
Krajnović et al. (2016)	48	3	15,00	1.15	3	15.77	1.74	Human melanoma	A375	Xanthohumol
Krajnović et al. (2016)	48	3	22.15	4.05	3	21.88	5.19	Murine melanoma	B16	Isoxanthohumol
Krajnović et al. (2016)	48	3	22.90	0.78	3	24.18	1.43	Human melanoma	A375	Isoxanthohumol
Krajnović et al. (2019)	48	3	30.00	4.05	3	42.00	5.19	Murine melanoma	B16-F10	Isoxanthohumol
Krajnović et al. (2016)	48	3	40.85	0.78	3	38.55	8.84	Murine melanoma	B16	8-prenylnaringenin
Krajnović et al. (2016)	48	3	27.80	3.82	3	48.30	11.6	Human melanoma	A375	8-prenylnaringenin
