# Supplementary material for: Humulus lupulus (Hop)-Derived Chemical Compounds Present Antiproliferative Activity on Various Cancer Cell Types: A Meta-Regression Based Panoramic Meta-Analysis
Source: Pharmaceuticals (Basel). 2025 Jul 31;18(8):1139. doi: 10.3390/ph18081139 (PMC12388921; doi:10.3390/ph18081139)
Supplement: Supplementary file 1 [file pharmaceuticals-18-01139-s001.zip › SUP_TABLE 5.pdf]

Supplementary Table S5. Random effects meta-analysis of IC_{50} values of Xanthohumol on different types of cancer and non-cancer cells for different incubation time points.

Type of cancer	Time	Number of studies	IC_{50}(μM)	95% CI	p-value
Glioblastoma	24	5	60.44	55.13, 65.75	0.000
Neck	24	2	50.80	40.69, 60.99	0.000
Gastric	24	4	42.75	0.00, 88.89	0.069
Liver	24	6	88.08	52.80, 123.35	0.000
Pancreas	24	2	18.85	1.50, 36.20	0.033
Colon	24	5	33.83	11.56, 56.11	0.003
Breast	24	7	48.97	29.38, 68.57	0.000
**Cancer**	**24**	**37**	**52.16**	**42.66, 61.66**	**0.000**
**Non-cancer**	**24**	**9**	**90.03**	**35.94, 144.11**	**0.001**
Leukemia	48	3	15.22	9.55, 20.89	0.000
Myeloma	48	2	35.99	8.28, 63.69	0.011
Melanoma	48	5	12.54	8.89, 16.20	0.000
Neck	48	4	18.93	14.11, 23.74	0.000
Lung	48	3	15.50	3.18, 27.81	0.014
Liver	48	3	11.00	6.68, 15.31	0.000
Pancreas	48	2	8.600	2.76, 14.44	0.004
Colon	48	6	17.02	8.62, 25.41	0.000
Breast	48	7	21.75	13.26, 30.24	0.000
Cervix	48	2	21.27	0.00, 46.85	0.103
Ovarian	48	2	8.26	0.00, 23.43	0.286
Prostate	48	3	13.86	11.36, 16.37	0.000
**Cancer**	**48**	**46**	**17.96**	**15.19, 20.73**	**0.000**
**Non-cancer**	**48**	**12**	**59.53**	**47.78, 71.27**	**0.000**
Leukemia	72	3	6.58	3.26, 9.91	0.000
Neck	72	2	18.65	12.81, 24.49	0.000
Lung	72	2	17.02	9.82, 24.21	0.000
Liver	72	4	39.79	0.00, 83.02	0.071
Pancreas	72	5	11.86	7.16, 16.56	0.000
Colon	72	8	23.45	19.48, 27.43	0.000
Breast	72	13	11.60	10.10, 13.09	0.000
Ovarian	72	2	5.14	0.00, 11.17	0.095
Prostate	72	9	13.03	10.61, 15.45	0.000
**Cancer**	**72**	**52**	**16.89**	**15.18, 18.60**	**0.000**
**Non-cancer**	**72**	**9**	**31.80**	**10.31, 53.29**	**0.004**

"Cancer" denotes meta-analysis results for collectively all cancer cell lines
