# Supplementary material for: Humulus lupulus (Hop)-Derived Chemical Compounds Present Antiproliferative Activity on Various Cancer Cell Types: A Meta-Regression Based Panoramic Meta-Analysis
Source: Pharmaceuticals (Basel). 2025 Jul 31;18(8):1139. doi: 10.3390/ph18081139 (PMC12388921; doi:10.3390/ph18081139)
Supplement: Supplementary file 1 [file pharmaceuticals-18-01139-s001.zip › SUP_TABLE 6.pdf]

Supplementary Table S6. Meta-regression analysis of IC_{50} values of all chemical compounds on cancer and non-cancer cells for all incubation time points.

	**p-value**	**Number of studies**	**Compound**
Cancer	0.000	262	All
Non-cancer	0.038	55	All
Cancer	0.000	141	Xanthohumol
Non-cancer	0.040	31	Xanthohumol
Cancer	0.224	42	Isoxanthohumol
Cancer	0.005	26	8-prenylnaringenin
Cancer	0.588	11	6-prenylnaringenin
Cancer	0.301	6	Lupulone

"Cancer" denotes meta-analysis results for collectively all cancer cell lines
