# Supplementary figures and images for: Humulus lupulus (Hop)-Derived Chemical Compounds Present Antiproliferative Activity on Various Cancer Cell Types: A Meta-Regression Based Panoramic Meta-Analysis
Source: Pharmaceuticals (Basel). 2025 Jul 31;18(8):1139. doi: 10.3390/ph18081139 (PMC12388921; doi:10.3390/ph18081139)

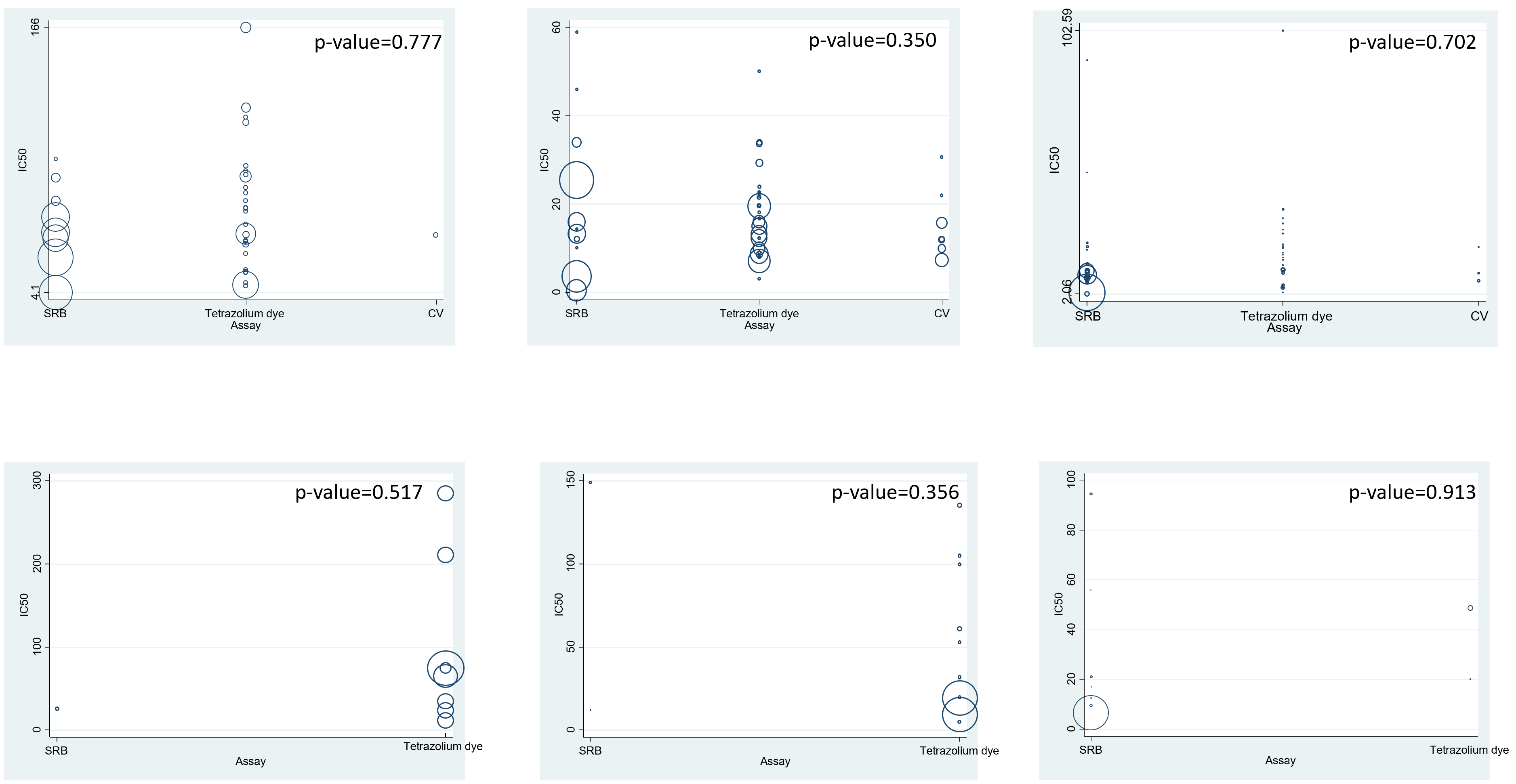

Supplement: Supplementary file 1 [file pharmaceuticals-18-01139-s001.zip › Supplementary Figure 1.tif]

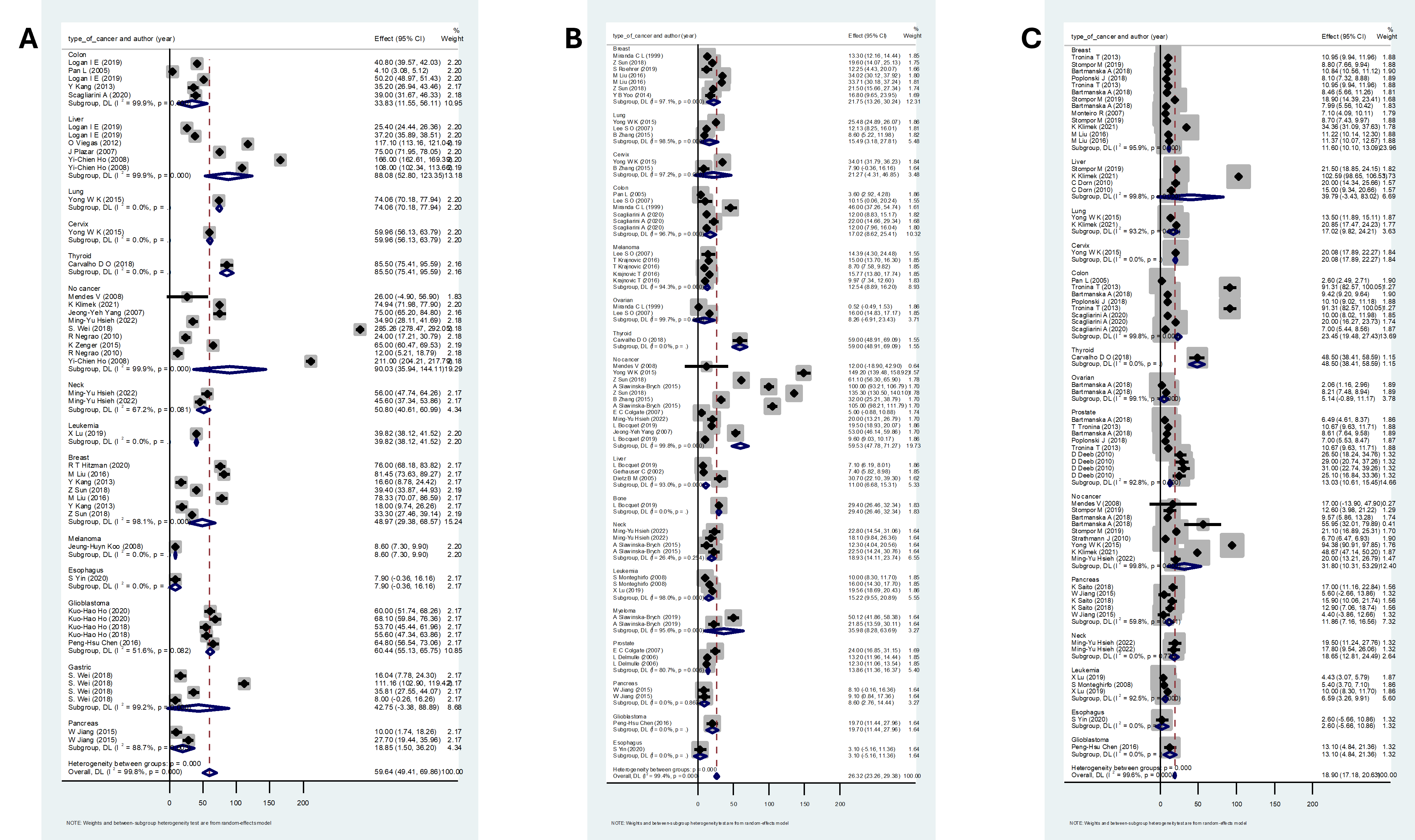

Supplement: Supplementary file 1 [file pharmaceuticals-18-01139-s001.zip › Supplementary Figure 2.tif]

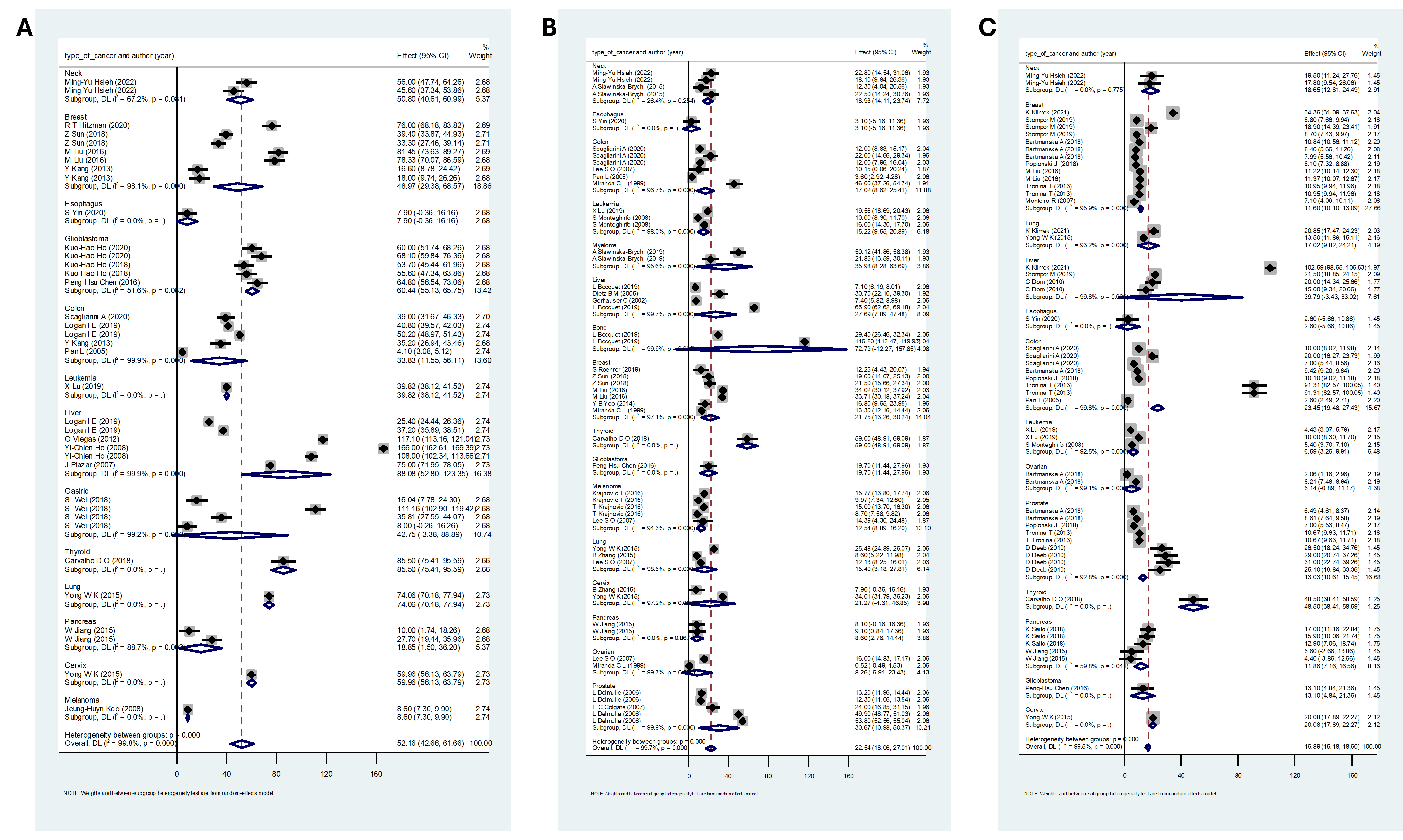

Supplement: Supplementary file 1 [file pharmaceuticals-18-01139-s001.zip › Supplementary Figure 3_ABC.tif]

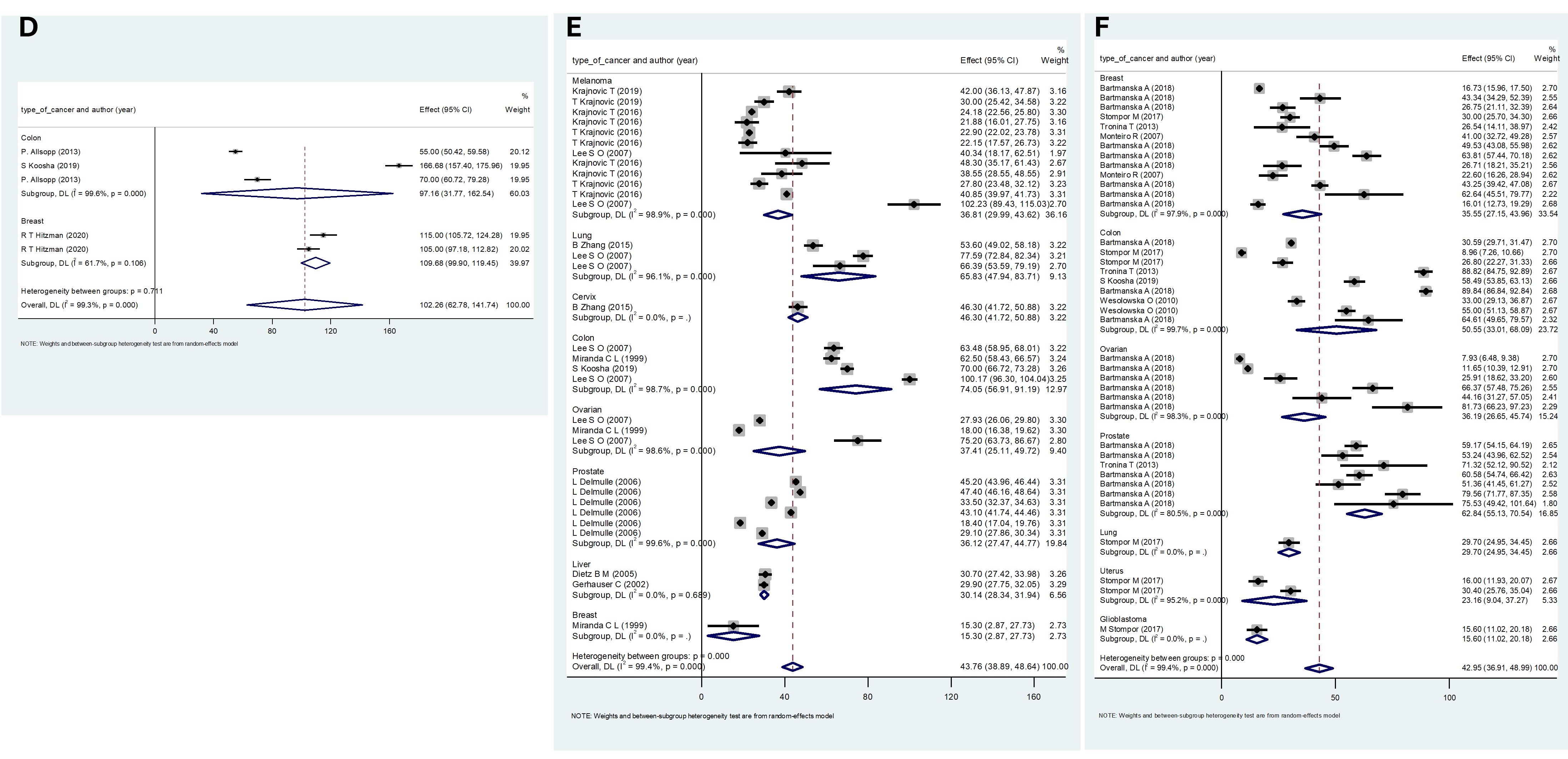

Supplement: Supplementary file 1 [file pharmaceuticals-18-01139-s001.zip › Supplementary Figure 3_DEF.png.tif]
